# Sphingosine-1-Phosphate Catabolizing Enzymes Predict Better Prognosis in Triple-Negative Breast Cancer Patients and Correlates With Tumor-Infiltrating Immune Cells

**DOI:** 10.3389/fmolb.2021.697922

**Published:** 2021-06-21

**Authors:** Rajeev Nema, Ashok Kumar

**Affiliations:** Department of Biochemistry, All India Institute of Medical Sciences (AIIMS) Bhopal, Bhopal, India

**Keywords:** breast cancer, sphingosine-1-phosphate, SGPP1, PLPP3, S1PR4, tumor-infiltrating immune cells

## Abstract

**Background:** Triple-negative breast cancer (TNBC) is associated with a poor prognosis. Sphingosine-1-phosphate (S1P), a potent sphingolipid metabolite, has been implicated in many processes that are important for breast cancer (BC). S1P signaling regulates tumorigenesis, and response to chemotherapy and immunotherapy by affecting the trafficking, differentiation or effector function of tumor-infiltrating immune cells (TIICs).

**Objective:** In this study, using bioinformatics tools and publicly available databases, we have analyzed the prognostic value of S1P metabolizing genes and their correlation with TIICs in BC patients.

**Methods:** The expression of S1P metabolizing genes and receptors was evaluated by the UALCAN cancer database. The correlation between mRNA expression of S1P metabolizing genes and receptors and survival outcome of breast cancer patients was analyzed by the Kaplan-Meier plotter database. The association between the gene expression and infiltration of immune cells in the tumors was analyzed by “Tumor-Infiltrating Immune Estimation Resource (TIMER). *In silico* protein expression analysis was done using the Human Protein Atlas” database.

**Results:** TNBC patients with lower expression of S1P phosphatase 1 (*SGPP1*) or lipid phosphate phosphatase 3 (*PLPP3*) have much shorter relapse-free survival than the patients with a higher expression of these genes. *SGPP1* and *PLPP3* expression show a strong positive correlation with tumor-infiltrating dendritic cells (DCs), CD4+ and CD8+ T cells, neutrophils, and macrophages in the TNBC subtypes. In addition, S1P receptor 4 (S1PR4), an S1P receptor exhibit a strong positive correlation with DCs, CD4+ and CD8+ T cells and neutrophils in TNBC. We, therefore, conclude that low expression of *SGPP1* and *PLPP3* may hinder the recruitment of immune cells to the tumor environment, resulting in the blockage of cancer cell clearance and a subsequent poor prognosis.

## Introduction

Breast cancer is the foremost cause of cancer-related deaths in females in many countries, including India (Bray et al., 2018; Alkabban and Ferguson, 2020; Jonnada et al., 2020). Invasive BC is classified into four distinct subtypes based on the expression of estrogen receptor alpha (ERα), progesterone receptor (PR), human epidermal growth factor receptor 2 (HER2), and Ki-67 (Inic et al., 2014; Harbeck et al., 2019). The luminal A subtype (ERα+, PR+, HER2−and low expression of Ki-67) is a low-grade breast tumor, whereas the luminal B subtype (ERα+, PR+, HER2+/−and high expression of Ki-67) is a more aggressive form. The molecular subtypes of BC are often a key reference for prognosis and choice of therapeutic strategy (Inic et al., 2014; Harbeck et al., 2019). Triple-negative breast cancer (TNBC) (ERα−, PR− and HER2−) accounts for approximately 15% of all BC cases and is characterized by shorter survival and an early peak of distant recurrence (Manjunath and Choudhary, 2021). The term TNBC encompasses a highly diverse group of cancers that is further categorized into six molecular TNBC subtypes (Pietenpol subtypes), two basal-like subtypes (BL1 and BL2), one immunomodulatory (IM), one mesenchymal, one mesenchymal stem-like, and one luminal androgen receptor subtype (Lehmann et al., 2011). In the majority of cases, identifying TNBC response to traditional chemotherapy and immunotherapy poses a challenge in current clinical practice (Manjunath and Choudhary, 2021).

Tumor-infiltrating immune cells play an important role in cancer treatment efficacy and patient prognosis (Denkert et al., 2018). Further, TIICs, especially tumor-infiltrating lymphocytes (TILs), are associated with better outcomes in HER2+BC and TNBC. However, TNBC lacks both predictive markers and potential therapeutic targets. Hence the identification of novel predictive and prognostic markers is warranted. Sphingosine-1-phosphate (S1P) signaling has emerged as a central mediator of the trafficking of hematopoietic cells, including lymphocytes, natural killer (NK) cells, neutrophils, dendritic cells (DCs) and macrophages (Cartier and Hla, 2019; Pyne and Pyne, 2020). S1P activates several cellular pathways by binding to one of the five G-protein-coupled receptors (GPCRs), termed S1P receptors (S1PRs) (Sukocheva et al., 2006; Sukocheva, 2018). Activation of these receptors in an autocrine or paracrine manner by S1P promotes cell proliferation, cell survival migration, and activation of the inflammatory response and also inhibits apoptosis (Pyne and Pyne, 2020). Moreover, S1PRs are expressed on almost all the immune cell types. However, each cell type expresses only a subset of S1PRs. S1PR1 is expressed in most immune cells, whereas S1PR5 is expressed primarily by DCs and NK cells (Kumar et al., 2017).

S1P, an oncogenic sphingolipid (Fyrst and Saba, 2010), is generated by two isoforms of sphingosine kinase, *SPHK1* and *SPHK2* (Pyne and Pyne, 2020). S1P can be metabolized through irreversible cleavage by the S1P lyase enzyme (*SGPL1*) (Zhou and Saba, 1998) to *trans*-2-hexadecenal and ethanolamine phosphate. Alternatively, S1P can also be dephosphorylated back to sphingosine through a reaction that is catalyzed by three non-specific lipid phosphate phosphatases (LPP1–3) or two S1P-specific phosphatases (SPPase1–2) (Cartier and Hla, 2019; Pyne and Pyne, 2020). S1P-metabolizing enzymes play a crucial role in various aspects of BC, including neovascularization, metastasis, recurrence, and chemo resistance (Wang et al., 1999; Nagahashi et al., 2016). SphK1 has been shown to overexpress in breast tumor tissue compared to normal breast tissue, and higher expression of SphK1 is associated with poor survival outcomes (Ruckhäberle et al., 2008). SphK1 also promotes tumor angiogenesis, lymphangiogenesis, and resistance to radiation and chemotherapy (Sarkar et al., 2005; Pyne and Pyne, 2020). In a prior study, BC patients with lymph node metastasis showed significantly higher levels of S1P compared with BC patients with negative nodes (Tsuchida et al., 2017). However, the role of S1P-catabolizing enzymes, including SPPase1 and LPP3 (*PLPP3*), in BC is not yet fully understood.

The meta-analysis of publicly available data from The Cancer Genome Atlas (TCGA) databases can predict outcomes when applied to appropriately powered cohorts and is a feasible, unbiased approach that can be adapted to analyze the involvement of genes in cancer progression. Thus, in the present study, using web-based bioinformatics tools, the aim was to analyze the association of S1P-metabolizing enzymes and S1P receptors with TIICs in BC patients and with the prognosis of patients with BC. Expression of S1P-signaling genes in breast tumor and normal tissue by utilizing the UALCAN database (Chandrashekar et al., 2017) and the role of these genes in survival outcome was analyzed by employing the Kaplan-Meier (KM) plotter (Győrffy et al., 2013). To determine the role of S1P-metabolizing enzymes in the infiltration of immune cells into breast tumors, the Tumor Immune Estimation Resource (TIMER), an online bioinformatics tool for the comprehensive analysis of TIICs, was used (Li et al., 2017).

In this study, we demonstrate that the expression of *SGPP1* and *PLPP3* is reduced in tumors compared with normal tissues from patients with BC. Low expression of *SGPP1* and *PLPP3* in tumors could be considered to be a predictive marker for worse relapse-free survival (RFS) in patients with TNBC. We also found that both of these genes are associated with a high predictive value in systemically treated patients with BC. Importantly, we demonstrate that *SGPP1* and *PLPP3* expression is strongly associated with TIICs, especially DCs, CD8^+^ T cells, and neutrophils. Furthermore, we found that *S1PR4*, an S1P receptor, is strongly associated with TIICs in all four BC subtypes. Therefore, *SGPP1* and *PLPP3* could serve as predictive and prognostic markers in patients with TNBC.

## Materials and Methods

### Kaplan–Meier Survival Analysis

We analyzed the association between gene-specific mRNA expression and RFS of breast cancer patients by employing the KM plotter (www.kmplot.com) (Győrffy et al., 2013). The KM plotter is a web-based tool widely used in the meta-analysis of publicly available TCGA, gene expression omnibus, and European Genome-Phenome Archive databases (Győrffy et al., 2013). Currently, in the KM plotter, gene expression and survival outcome data for 3,955 patients with BC for a follow-up period of 20 years are available along with clinicopathological features such as ER, PR, and HER2 status, intrinsic subtypes, lymph node status, tumor grade, and Pietenpol subtypes as well as details of the systematic therapy (endocrine therapy or chemotherapy) given to patients (Szász et al., 2016). Data from 36 gene expression datasets was included for analysis by KM plotter, the details of datasets are given in Supplementary Material.

We analyzed the prognostic value of S1P-metabolizing enzymes and S1P receptors in breast cancer by entering their respective gene symbols into the KM plotter database (www.kmplot.com). The number of patients at-risk was indicated below the main KM plot. Affymetrix IDs of all the genes analyzed in the study are shown in Supplementary Table S1. The checkbox for auto-select best cut-off value was selected to divide the patients into high expression and low expression groups. JetSet probes showing concordance between protein measurements and gene expression values were used for survival analysis (Li et al., 2011). The hazard ratio (HR) with a 95% confidence interval (CI) and the log-rank *p*-value was calculated by the KM plotter (Wang et al., 2014). A *p* value <0.05 was considered statistically significant.

### Gene Expression Analysis Using UALCAN Database

UALCAN is a comprehensive, user-friendly, and interactive web resource that can be used to plot graphs depicting gene expression and to evaluate promoter DNA methylation information (Chandrashekar et al., 2017). In this study, it was also used for gene expression analysis of *SGPP1, SGPP2, PLPP1, PLPP2,* and *PLPP3* in breast tumors and normal tissues.

### Protein Expression Analysis Using Human Protein Atlas Database Tool

We also analyzed the protein expression of LPP3 and SGPP1, available in “the Human Protein Atlas” (HPA; https://www.proteinatlas.org/) database as described previously (Nema et al., 2021).

### Analysis of Tumor Immunological Features of S1P Components Using TIMER

The TIMER is a web-based computational tool for the evaluation of tumor immune cells in the publicly available TCGA database (Li et al., 2017). It uses a deconvolution method to deduce the abundance of TIICs based on gene expression profiles. It provides six major analytic modules that allow users to explore the associations between immune infiltrates and various factors, including clinical outcomes, somatic mutations, somatic copy number alterations, and gene expression (Li et al., 2017). In this study, the associations between the expression of S1P-metabolizing genes with TIICs in patients with BC, tumor-infiltrating immune infiltrate cells (B cells, CD4+ T cells, CD8+ T cells, macrophages, neutrophils, and DCs) were selected for correlation by TIMER. Tumor purity was taken into consideration when calculating the Spearman’s correlation. Because genes highly expressed in the infiltrating immune cells are expected to have negative associations with tumor purity. A *p* value <0.05 was considered statistically significant.

## Results

**FIGURE 1:**
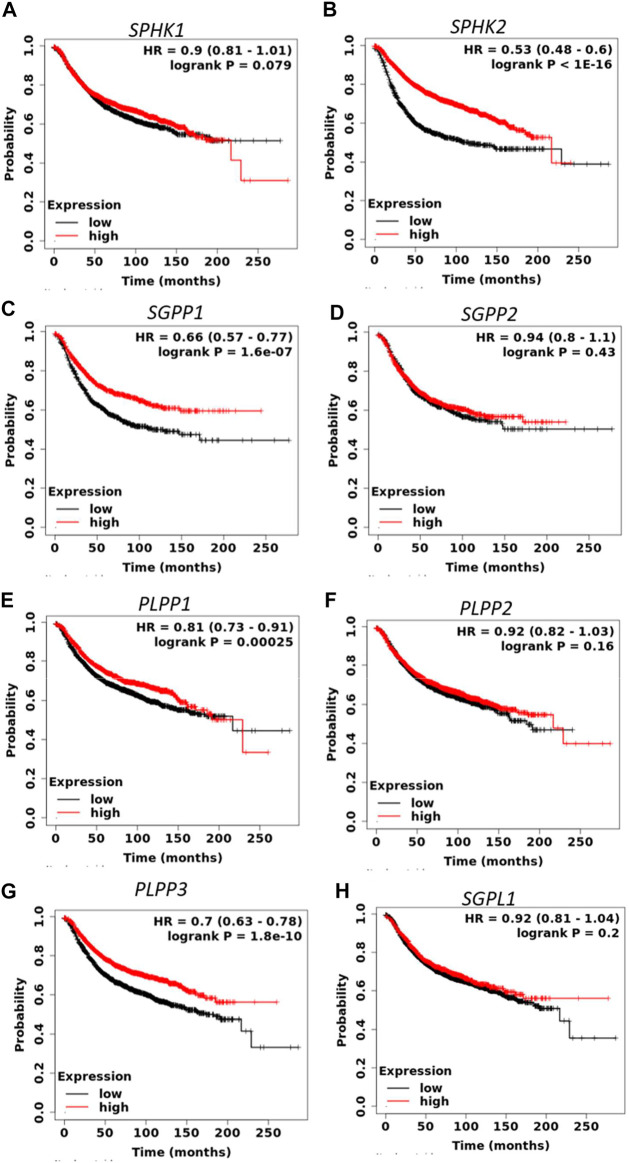
Prognostic role of mRNA expression of S1P-metabolizing enzymes **(A–H)** in breast cancer patients. Kaplan–Meier survival curves (RFS) were plotted for S1P-metabolizing genes from the publicly available KM plotter database (N = 3955 BC).

**FIGURE 2:**
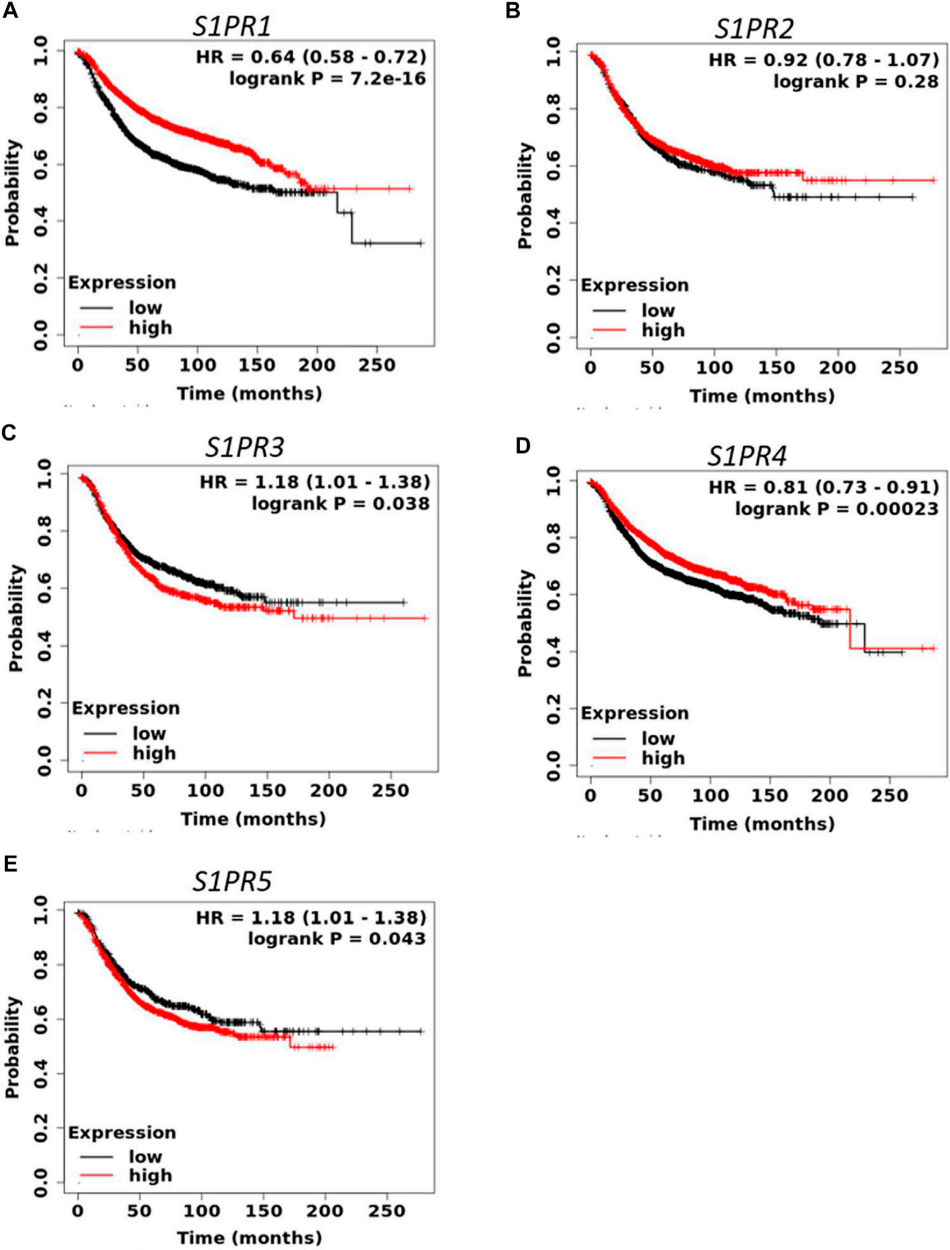
Prognostic role of mRNA expression of S1P receptors **(A–E)** in breast cancer patients. Kaplan-Meier survival curves (RFS) were plotted for S1P receptors from the publicly available KM plotter database (N = 3955 BC).

S1P signaling plays an important role in the tumorigenesis of a variety of cancers. To determine the prognostic value of S1P-metabolizing enzymes, KM survival curves were plotted for patients with BC for the mRNA expression of S1P-metabolizing enzymes. As shown in Figure 1 and Supplementary Table S2, mRNA expression of *SPHK2, SGPP1, PLPP1*, and *PLPP3* was significantly associated with RFS and overall survival (OS) in patients with BC. Of these, RFS was best predicted by *SPHK2* (P < 1e-16), *SGPP1* (*p* = 1.6e-07), and *PLPP3* (LPP3; *p* = 1.8e-10), where patients with BC with a lower expression of these enzymes had worse RFS (Figure 1). *SPHK1* and *SGPP2* did not show an appreciable significant association with the survival outcome of the patients with BC (Supplementary Table S2). The prognostic value of *SPHK2* in BC has been described previously (Alshaker et al., 2020). As *SGPP1* and *PLPP3* showed highly significant association with RFS as well as OS, we therefore focused our attention on *SGPP1* and *PLPP3*. S1P mediates its physiological functions by binding to S1P receptors. Thus, to predict the prognostic role of S1P receptors in relation to BC survival, KM plots were drafted for S1P receptors. As shown in Figure 2 and Supplementary Table S3, except for *S*1*PR*2, the expression of S1P receptors, *S*1*PR1*, *S*1*PR3*, *S*1*PR4*, and *S*1*PR5,* was significantly associated with RFS in patients with BC. Of these, high expression of *S*1*PR1* (HR = 0.64, 95% CI: 0.58–0.72, *p* = 7.2e-16) and *S*1*PR4* (HR = 0.81, 95% CI: 0.73–0.91, *p* = 0.00023) was significantly associated with better RFS in all patients with BC (Figure 2).

**FIGURE 3:**
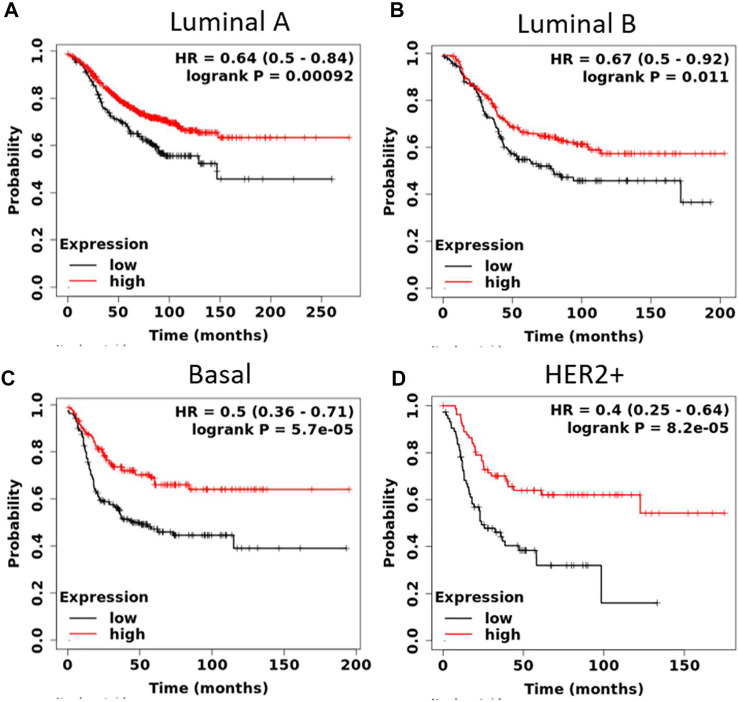
High expression of sphingosine-1-phosphate phosphatase1 (*SGPP1*) is highly significantly associated with relapse-free survival (RFS) of patients with HER2+ and basal-like BC (intrinsic subtypes). **(A**–**D)**, Kaplan-Meier survival curves (RFS) plotted for *SGPP1* for intrinsic subtypes of BC.

We also explored the prognostic value of *SGPP1* and *PLPP3* in BC patients with different intrinsic subtypes, pathological grades, and lymph node status. Interestingly, as shown in Figure 3 and Table 1, *SGPP1* showed a high predictive value in the basal and HER2+ subtypes. In the basal subtype, upper quartile survival in the low expression (*SGPP1*) cohort was 14.7 months compared to 30.42 months in the high expression (*SGPP1*) cohort (Figure 3C; Table 1). However, in the HER2+ subtype, upper quartile survival in the low expression (*SGPP1*) cohort was 12.0 months compared to 25.2 months in the high expression (*SGPP1*) cohort (Figure 3D). Among the Pietenpol subtypes of TNBC, despite having a low sample size, mesenchymal stem-like and luminal androgen receptor subtypes showed a significant association of *SGPP1* expression with RFS (Table 2). There was a five-fold difference in the survival period (RFS) between the *SGPP1* low expression cohort vs. the high expression cohort in the luminal androgen receptor subtypes of TNBC (Table 2), whereas *PLPP3* showed high predictive value in luminal A and basal subtypes (Table 1). *SGPP1* expression showed a distinct profile in PR + vs. PR-subtypes. Patients with BC with high expression of *SGPP1* had significantly better RFS in the PR + subtype (Table 3 Supplementary Figure S1) compared to the PR-subtype (Table 3; Supplementary Figure S1). However, *PLPP3* showed a distinct RFS profile in ER+ vs. ER− subtypes. *SGPP1* also exhibited a significant association in lymph node (LN)+ patients with BC, but not in LN− patients (Table 3; HR = 0.65, 95% CI: 0.5–0.84, *p* = 0.0011).

**TABLE 1:** Correlation between mRNA expression of *SGPP1* and *PLPP3* with relapse-free survival in the different breast cancer intrinsic subtypes.

Gene	Intrinsic subtype	No. of patients	Hazard ratio	*p* value	RFS (in months)	
					Low expression cohort	High expression cohort
SGPP1	Luminal A	841	0.64	0.00092	39.0	66.5
	Luminal B	407	0.67	0.011	30.0	39.0
	HER2+	156	0.40	8.2e-05	12.0	25.2
	Basal	360	0.50	5.7e-05	14.7	30.4
*PLPP3*	Luminal A	1,933	0.68	1.2e-05	60.0	97.0
	Luminal B	1,149	0.73	0.0024	161.3	171.4
	HER2+	251	0.60	0.0092	15.0	25.0
	Basal	618	0.59	8.7e-05	13.0	25.0

**TABLE 2:** Correlation between mRNA expression of *SGPP1* and *PLPP3* with relapse-free survival (RFS) in Pietenpol subtypes of triple-negative breast cancer (TNBC).

S.No	TNBC subtypes	HR	Sample size N	*p* value	RFS	
					Low expression cohort	High expression cohort
*SGPP1*						
1	Basal-like (BL1)	0.57	105	0.06	12	29
2	Basal-like (BL2)	0.66	52	0.33	47.28	122.64
3	Immunomodulatory (IM)	1.61	110	0.38	NA	NA
4	Mesenchymal (M)	0.55	101	0.086	13	14.64
5	Mesenchymal stem–Like (MSL)	0.39	33	0.063	15.25	34
6	Luminal androgen receptor (LAR)	0.45	100	0.026	19.23	98.4
PLPP3						
1	Basal-like (BL1)	0.51	171	0.0052	11.07	29
2	Basal-like (BL2)	0.65	76	0.32	18.9	21
3	Immunomodulatory (IM)	0.64	203	0.13	55.66	114.96
4	Mesenchymal (M)	0.64	177	0.53	10.62	18.6
5	Mesenchymal stem–Like (MSL)	0.22	63	6.9 × 10–5	14.86	74
6	Luminal androgen receptor (LAR)	0.49	203	0.00058	14	25

**TABLE 3:** Correlation between mRNA expression of *SGPP1* and *PLPP3* with relapse-free survival in the breast cancer patients with ER, PR, HER2 and LN status.

Gene	ER/PR/HER2/LN status	Patients	Hazard ratio	95% CI	*p* value
*SGPP1*	ER + ve	762	0.71	0.52–0.96	0.026
	ER -ve	347	0.70	0.5–0.98	0.037
	PR + ve	489	0.61	0.37–1	0.046
	PR -ve	372	0.73	0.5–1.05	0.088
	HER2 +ve	150	0.56	0.29–1.07	0.074
	HER2 -ve	635	0.73	0.53–1	0.05
	LN + ve	724	0.65	0.5–0.84	0.0011
	LN -ve	496	0.64	0.39–1.03	0.062
*PLPP3*	ER + ve	2061	0.78	0.65–0.92	0.0034
	ER -ve	762	0.90	0.72–1.12	0.34
	PR + ve	589	0.8	0.57–1.15	0.23
	PR -ve	549	1.34	0.95–1.9	0.098
	HER2 +ve	252	1.66	1.07–2.57	0.021
	HER2 -ve	800	0.82	0.62–1.09	0.17
	LN + ve	1,133	0.78	0.64–0.95	0.015
	LN -ve	2020	0.75	0.63–0.90	0.0015

To analyze whether *SGPP1* and *PLPP3* could serve as a predictive marker for response to systemic therapy, an RFS curve was plotted for the intrinsic subtypes of patients who had received any kind of systemic therapy (endocrine or chemotherapy). Both of the S1P-catabolizing genes, *SGPP1* and *PLPP3,* showed high predictive value for response to systematic therapy in invasive breast carcinoma patients and especially in the HER2+ and basal subtypes (Table 4). Basal subtypes treated with any type of systemic therapy and with higher expression of *SGPP1* or *PLPP3* had almost four-fold (*SGPP1*) and 2.5-fold (*PLPP3*) RFS compared to the low expression cohort (Table 4). A multivariate analysis of *SGPP1* and *PLPP3* with selected variables such as MKI67, ESR1 and ERB2 showed a highly significant association with RFS (Supplementary Figure S2).

**TABLE 4:** Correlation between mRNA expression of *SGPP1* and *PLPP3* with relapse-free survival (RFS) in the systemically treated in different breast cancer intrinsic subtypes.

S.No	Subtypes	HR	No. of patients	*p* value	Upper quartile RFS months	
					Low expression cohort	High expression cohort
*SGPP1*						
1	All	0.69	751	0.00097	60	113.8
2	Luminal A	0.62	305	0.014	30	42
3	Luminal B	0.77	176	0.2	44	55.4
4	HER2+	0.45	76	0.024	12.3	20
5	Basal	0.55	194	0.0038	29	115
*PLPP3*						
1	All	0.65	1881	2.9e-07	35	58.15
2	Luminal A	0.71	861	0.012	64.9	97.3
3	Luminal B	0.69	596	0.0063	110.3	171.43
4	HER2+	0.4	125	0.005	16.0	58.15
5	Basal	0.43	299	2.2e-06	10.32	25

### Expression of *SGPP1* and *PLPP3* in Primary Breast Tumors is Decreased

**FIGURE 4:**
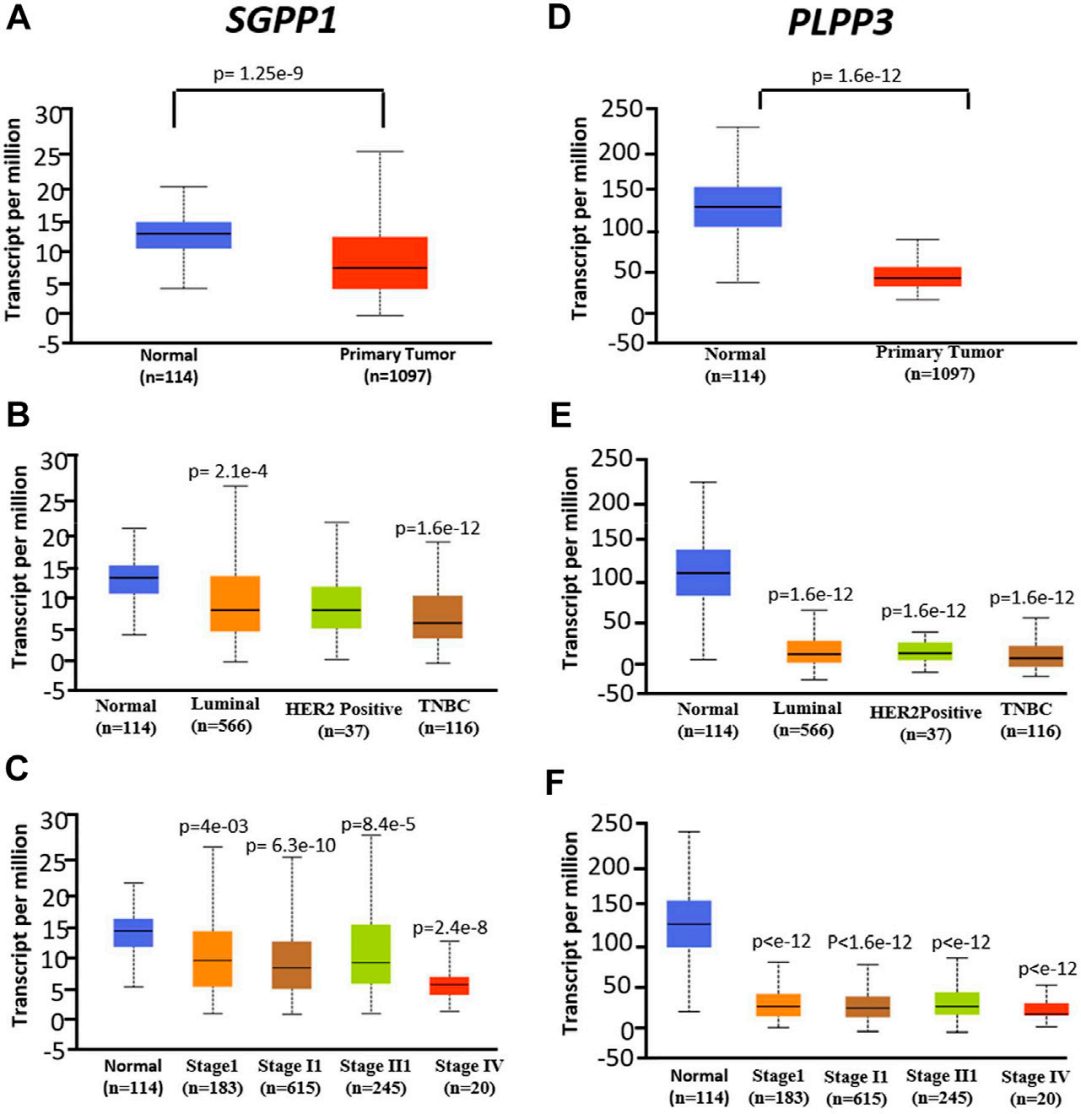
mRNA expression was analyzed in normal tissue (N = 114) and primary tumors from breast cancer (N = 1,097) patients from the publicly available UALCAN database. **(A–C)**, *SGPP1* (where A = normal vs. tumors, B = normal vs. cancer subtypes and C = normal vs. tumors of different stages). *p < 0.01 Stage I vs Stage IV, Stage II vs Stage IV and Stage III vs Stage IV. **(D–F)**, *PLPP3* (where D = normal vs. tumors, E = normal vs. cancer subtypes and F = normal vs. tumors of different stages). Data are shown as average number of transcripts per million. *p* values are calculated for primary tumors vs. normal tissues in corresponding genes.

mRNA expression of *SGPP1* and *PLPP3* genes in invasive cancer and normal breast tissues was compared using the UALCAN database (Chandrashekar et al., 2017). Compared to normal tissue, mRNA expression of *SGPP1* and *PLPP3* was decreased approximately 50 and 75% (*SGPP1 p* = 1.29 × 10^−9^; *PLPP3 p* = 1.29 × 10^−12^), respectively, in primary tumors from patients with BC (Figure 4A). Among the major subtypes of BC, patients with TNBC showed a pronounced decrease in *SGPP1* mRNA expression compared to luminal and HER2+ patients (Figure 4B). The decrease in the *SGPP1* and *PLPP3* mRNA expression was even more pronounced in the advanced stage (stage IV) (Figures 4C–F). Expression of the second isoform of SGPP i.e., *SGPP2*, was not significantly different in breast invasive carcinoma compared to normal tissue (Supplementary Figure S3A). However, the expression of *PLPP1* and *PLPP2* was decreased and increased, respectively, in primary breast tumors compared to normal breast tissue (Supplementary Figures S3B,C).

Gene expression is regulated by several epigenetic factors, including DNA methylation. Therefore, we used the UALCAN database to verify the methylation status of the *SGPP1* gene promoter. As shown in Supplementary Figures S4A–C, an increase in *SGPP1* promoter methylation status was noted in patients with TNBC; however, no significant difference in the methylation status of the *SGPP1* promoter was observed in the other BC subtypes (luminal and HER2+) vs. normal subjects. Notably, the promoter of *PLPP3* was hypermethylated in the primary tumors from invasive breast carcinoma (Supplementary Figures S4D–F). Furthermore, hypermethylation of the *PLPP3* was more pronounced in TNBC subtypes than luminal subtypes (*p* = 0.0139) (Supplementary Figure S4E).

Following on from the above, we analysed SGPP1 and PLPP3 protein expression in breast tumors and normal breast tissue by using The Human Protein Atlas (HPA; https://www.proteinatlas.org/) database. The results showed that the protein levels of PLPP3 and SGPP1 were decreased significantly in primary breast tumors as compared to normal breast tissue (Supplementary Figure S5). Furthermore, the IRS score (IHC) revealed that PLPP3 was expressed at a low level, and SGPP1 was moderately expressed in a patient with BC (Supplementary Figure S5).

### Somatic Mutations in S1P-Metabolizing Genes and S1P Receptors Are Uncommon

Mutation analysis of S1P-metabolizing genes and S1P receptors was performed by applying the cBioPortal (www.cbioportal.org) tool to the publicly available whole-exome sequencing data of patients with BC (Gao et al., 2013). Except for *SPHK1*, S1P-metabolizing genes were infrequently mutated in patients with BC (Supplementary Figure S6A). Among these, the *SPHK1* and *SGPL1* genes were mutated in 5 and 2.0% of patients with BC, respectively, where most of these alterations were due to gene amplification. Mutations, mostly deep deletions in the *PLPP3* gene in 1.2% of patients with BC, were noted (Supplementary Figure S6A). The cBioPortal was also used to analyze somatic mutations in the gene coding for S1P receptors in patients with BC. Among the S1P receptors, *S*1*PR2* and *S*1*PR5* were mutated in 1.6% and 1.8%of patients with BC, respectively (Supplementary Figure S6B).

### SGPP1 Expression Correlates With Infiltration of DCs and CD8+ Cytotoxic Cells in TNBC

**FIGURE 5:**
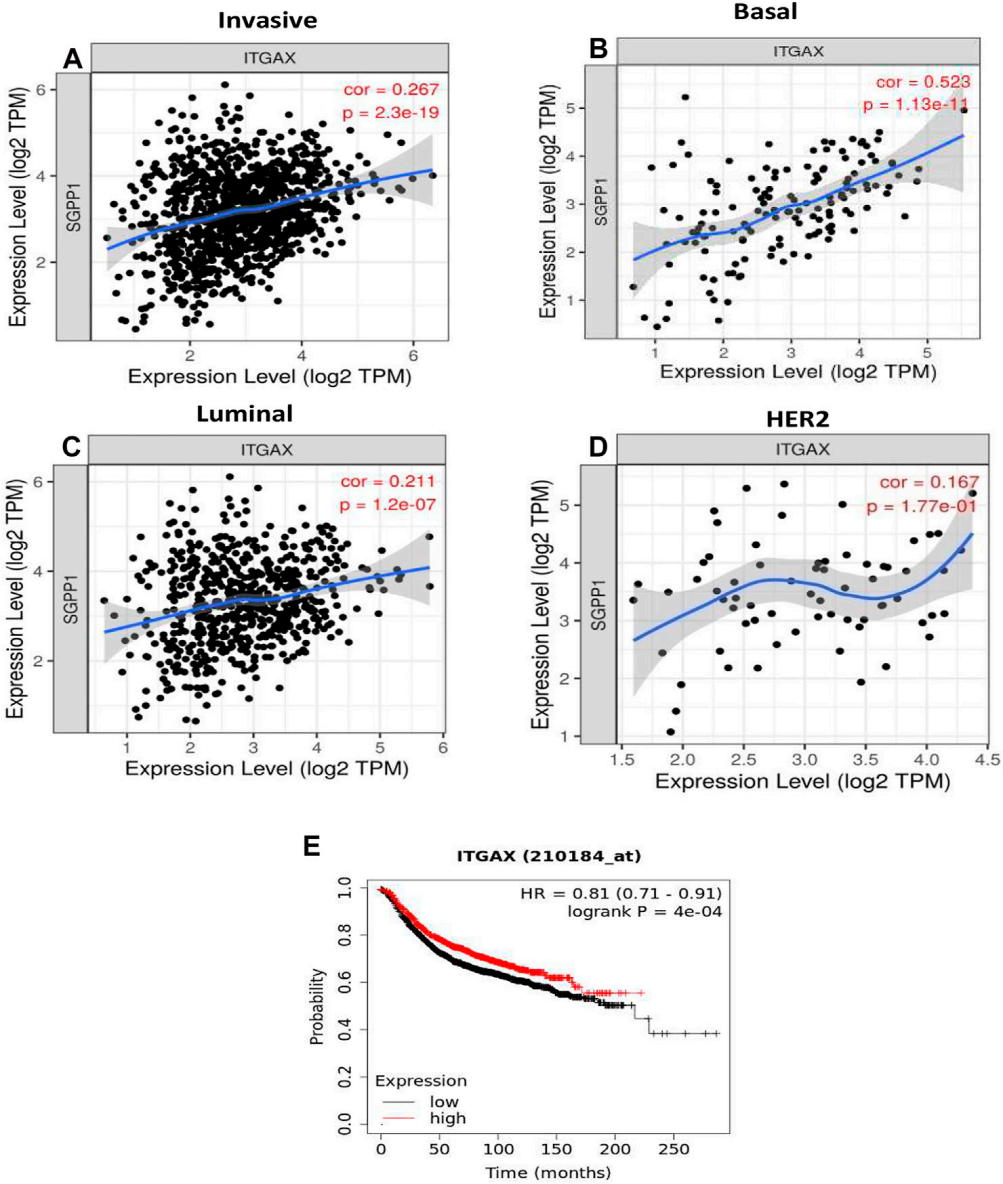
The *SGPP1* gene with invasive breast carcinoma, basal, luminal and HER2+ and *SGPP1*is significantly associated with basal-like tumor-infiltrating immune cells according to correlation via TIMER. **(A–D)**, *SGPP1* (where A = invasive breast carcinoma, B = basal-like breast carcinoma, C = luminal breast carcinoma, and D = HER2+ breast carcinoma). High expression of integrin subunit alpha X (*ITGAX*) is significantly associated with relapse-free survival (RFS) of breast cancer patients. **(E),** Kaplan-Meier survival curves (RFS) were plotted for *ITGAX* for breast cancer.

The role of the S1P-signaling pathway in the regulation of the infiltration of immune cells into tumor stroma in BC has not yet been elucidated. CD11c (integrin subunit alpha X; ITGAX), a member of the integrin β2 adhesion molecule family, is highly expressed in myeloid DCs. Conventional macrophages and DCs are crucial for antigen presentation and T-cell activation during antitumor immunity. Infiltration of DCs into the tumor microenvironment, enhances immune activation and recruitment of effector T cells (Janco et al., 2015). Therefore, the correlation of the mRNA expression of S1P-metabolizing enzymes with TIICs were analyzed using TIMER on publicly available TCGA datasets. A strong positive correlation (ρ = 0.523; *p* = 1.13e-13) was found form RNA expression of *SGPP1* and *ITGAX* (CD11c), a marker for DCs in TNBC or basal-like BC (Figures 5A–D). The mRNA expression of *SGPP1* was also associated with tumor DCs in the other cancer types. TNBC (basal-like BC) showed the highest correlation, followed by uveal melanoma, ovarian cancer, and thymomas (Supplementary Table S4). Moreover, survival curve analysis revealed that BC patients with high expression of CD11c had better survival outcomes (Figure 5E). Furthermore, in TNBC, expression of *SGPP1* was also correlated with TILs (CD8+ and CD4+ T cells), macrophages, and neutrophils (Supplementary Figure S7). As regards *PLPP3* and *SPHK1*, their expression exhibited a moderate positive correlation with TIICs except for the B cells in the luminal subtype, but not in the other subtypes of BC (Supplementary Figures S8, S9). As for *PLPP1*, its expression showed a moderate positive correlation with the infiltration of CD8+ T lymphocytes into the tumors of luminal subtypes (Supplementary Figure S10).

### Expression of S1PR4 Correlates With Tumor Infiltration of Immune Cells in BC

**FIGURE 6:**
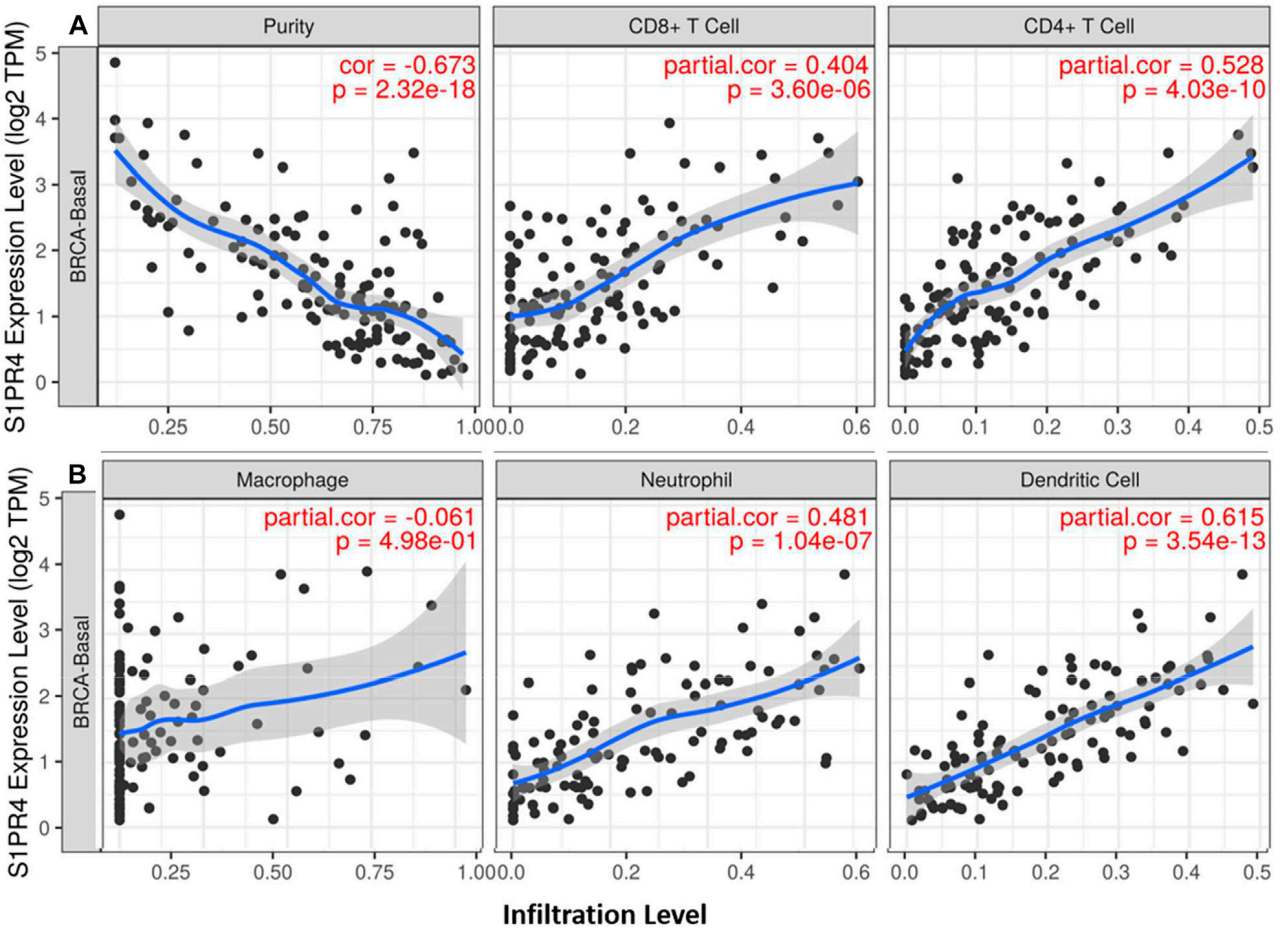
Correlation of *S*1*PR4* expression with immune infiltration level in breast cancer patients. *SGPP1* expression is significantly correlated with infiltrating levels of CD8+ T cells, CD4+ T cells **(A)**, neutrophils and dendritic cells in basal-like BC, but not with macrophage cells **(B)**.

Expression of S1P receptor(s) on immune cells is essential for their transport among various tissues, including lymph nodes, bone marrow, and tumor tissues (Rivera et al., 2008). Different types of immune cells express different types of S1P receptors. Therefore, to ascertain the role of S1P receptors in TIICs, the correlation between the expression profile of S1P receptors and immune cell markers was determined. Among all the S1P receptors, *S*1*PR4* showed the strongest correlation with TIICs, except for macrophages, in all the subtypes of BC (Figure 6). *S*1*PR1* showed moderate to strong correlation with TIICs, except for B cells, in all BC subtypes (Supplementary Figures S11, S12). *S*1*PR2* exhibited a moderate to strong correlation with TIICs in luminal and HER2+ subtypes (Supplementary Figure S13).

## Discussion

The difficulty of developing effective therapies for TNBC can be attributed to the high level of heterogeneity in the tumors. Hence, the identification of novel prognostic markers would help in stratifying patients for immunotherapy or targeted drug therapy. S1P signalling regulates various aspects of tumorigenesis, including proliferation, survival, invasion, angiogenesis, metastasis, and recurrence as well as patient response to chemotherapy and adjuvant therapy for various cancers, including BC (Alshaker et al., 2020). S1P mediates its diverse actions by binding to its five S1P receptors that belong to the GPCR family of receptors. Each S1P receptor is coupled to a specific G-protein and effector enzyme, resulting in the activation of various downstream signaling proteins, including Rac, Rho, Akt, and STAT3 (Postma et al., 1996; Zondag et al., 1998; Pyne and Pyne, 2020). Each cell type may express one or more S1P receptors at a given time, depending on the specificity and diversity of S1P functions. SphK1 is a well-studied kinase, responsible for S1P synthesis. It is overexpressed in the tumor tissues of patients with BC and thus serves as a marker for poor prognosis (Pyne and Pyne, 2020).

Using web-based bioinformatics tools, we found that patients with invasive BC and with lower expression of *SPHK2*, *SGPP1,* and *PLPP3* in primary tumors had worse RFS and OS. Our findings on *SPHK2* and *PLPP3* corroborate those in a previous report (Lei et al., 2018). In the present study, *SGPP1* had a high predictive value in the HER2+ and basal (TNBC) subtypes of BC, whereas *PLPP3* had a high predictive value in luminal A and basal subtypes. Furthermore, we found that the prognostic value of *SGPP1* also differed among the Pietenpol subtypes of TNBC (Lehmann et al., 2011). In particular, the difference in RFS in BL2 and IM subtypes was remarkable with differential gene expression of *SGPP1* and *PLPP3.*

Our results also revealed that expression of the catabolizing enzymes *SGPP1* and *PLPP3* decreased in primary tumors compared to normal breast tissues, and the reduction in the expression was notable in TNBC subtypes and stage IV patients. Furthermore, in our study, a decrease in the mRNA expression of *PLPP3* in breast tumors was found to be associated with the promoter hypermethylation, whereas no considerable change in the promoter methylation was observed for *SGPP1*. Decreased expression of S1P-catabolizing enzymes in tumors may lead to elevated levels of S1P in tumor tissues. Indeed, significantly higher levels of S1P have been reported in tumor tissues from BC patients with lymph node metastasis (Tsuchida et al., 2016).

Ectopic expression of mouse *SGPP1* in NIH3T3 fibroblasts depletes S1P levels and increases ceramide levels, thereby inducing apoptosis (Mandala, 2001). The small interfering RNA (siRNA) knockdown of endogenous *SGPP1* showed the accumulation of S1P within cells and significantly increased the secretion of S1P into the media, suggesting that *SGPP1* regulates the secretion of S1P into the extracellular milieu (Olivera et al., 2013). Furthermore, siRNA-induced knockdown of *SGPP1* in MCF-7 cells conferred resistance to tumor necrosis factor-α and the chemotherapeutic agent, daunorubicin (Johnson et al., 2003). SGPP1 is downregulated in gastric cancer and plays a role in invasion and migration in gastric cancer cells (Gao et al., 2015). *SGPP1* has also been shown to be a target of miR-95, which is highly expressed in ALDH+ and CD133+ subpopulations of non-small cell lung cancer (NSCLC) compared to ALDH1- and CD133-cells. miR-95 overexpression confers radio-resistance to ALDH1+ and CD133 + NSCLC by downregulating *SGPP*1 (Helleman et al., 2006; Huang et al., 2013). Our study demonstrated that SGPP1 expression positively correlates with better survival outcomes in systemically treated patients with BC, particularly in patients with TNBC. The mechanism by which *SGPP1* is decreased in the tumors of BC patients is not fully understood. However, hypermethylation of the *SGPP1* promoter does not seem to be involved in the repression of its gene expression. An earlier study has shown that transcriptional repressor GFI1 negatively regulates the expression of SGPP1 and that GFI1-dependent SGPP1 repression promotes growth and survival of multiple myeloma cells (Schwiebs et al., 2017).

The active sites of LPPs are present in the outer leaflet of the plasma membrane (Benesch et al., 2016); thus LPPs are able to degrade S1P, LPA, and other phospholipids present in the extracellular environment. LPP3 expression is downregulated in several cancer lines under hypoxic conditions (Harper et al., 2019), and its downregulation has also been observed in lung biopsies from patients with NSCLC (Magkrioti et al., 2018; Nema et al., 2021). We have also shown that the expression of the gene coding for LPPs, particularly LPP3 *(PLPP3),* is downregulated in the tumor tissues from oral squamous cell carcinoma patients compared to adjacent normal tissues (Vishwakarma et al., 2017).

The tumor microenvironment is controlled by the complex interaction of tumor cells with immune cells, blood vessels and cancer-associated fibroblasts. S1P signaling through its receptors regulates the migration of immune cells between lymphoid organs, circulation and peripheral tissues (Cartier and Hla, 2019; Pyne and Pyne, 2020). In the tumor microenvironment of patients with TNBC, CD11c positivity correlates with CD4+ and CD8+ T cells. Optimal immune response to tumor cells requires cytotoxic and Th1 cell response induced by antigen-presenting cells. In this context, the capacity of S1P to impair the differentiation of DCs from peripheral monocytes may contribute to the progression of carcinogenesis. Furthermore, a prior study has shown that SGPP1 is localized in the nucleus of naive DCs, and when SGPP1 encounters inflammatory stimuli, SGPP1 translocates into the endoplasmic reticulum and gets activated, resulting in the dephosphorylation of S1P (Schwiebs et al., 2017).

Activation of S1P signaling through S1PR1 is associated with carcinogenesis, metastasis. However, few studies have shown the decrease of S1PR1 in the cell line from triple-negative breast cancer (Abuhussein and Yang, 2020). Furthermore, in a mouse model of lung metastasis, compared to wild type, mice lacking S1pr1 in vascular endothelium (S1pr1 ECKO) developed excessive vascular sprouting and branching, and decreased barrier function, S1pr1 ECKO also developed larger tumors, and enhanced lung metastasis (Cartier et al., 2020). Thus, S1PR1 may exhibit tumor-suppressive attributes in the different cancers in the different histological subtypes or stage-specific manner. The discussion section of the manuscript has been revised accordingly. Expression of S1PR4 is restricted to lymphoid and hematopoietic tissues, and it has been shown previously that *S*1*PR4-*deficient mice have reduced numbers of plasmacytoid DCs (pDCs), which has been partly attributed to defects in the differentiation of pDC progenitors into mature pDCs (Dillmann et al., 2015). Infiltration of immune cells, including CD4+ and CD8+ T lymphocytes and DCs into tumors, is associated with better survival of patients with TNBC (Chao et al., 2020). We demonstrated herein that the expression of *SGPP1*, *PLPP3,* and S1P receptors, particularly S1PR4, positively correlates with tumor-infiltrating DCs in TNBC.

In summary, we found that low mRNA expression of *SGPP1* and *PLPP3* correlates with worse prognosis in patients with BC, particularly in those with TNBC, and that high expression of both of these genes may promote the infiltration of immune cells, especially DCs, into the tumor microenvironment (Supplementary Figure S14). Taken together, our findings indicate that *SGPP1* may serve as a potential prognostic marker that may be used to predict the effectiveness of systemic therapy in TNBC patients. However, we understand the limitation of our study as our findings are based on the datamining analysis carried out with multiple bioinformatics web-based tools. The prognostic markers identified in the current study need to be validated further in the wet lab. Experimental validation of these findings will be critical in further understanding the interplay between the S1P-signaling pathway in the TIICs and the prognosis of patients with BC.

## Data Availability

The original contributions presented in the study are included in the article/Supplementary Material, further inquiries can be directed to the corresponding author.
