# Supplementary material for: Sphingosine-1-Phosphate Catabolizing Enzymes Predict Better Prognosis in Triple-Negative Breast Cancer Patients and Correlates With Tumor-Infiltrating Immune Cells
Source: Front Mol Biosci. 2021 Jun 21;8:697922. doi: 10.3389/fmolb.2021.697922 (PMC8255376; doi:10.3389/fmolb.2021.697922)
Supplement: Supplementary file 1 [file DataSheet1.docx]

**Supplementary Material**

**Title: Sphingosine-1-Phosphate catabolizing enzymespredict better prognosis in triple-negative breast cancer patients correlate with tumor-infiltrating immune cells**

**Authors:** Rajeev Nema and Ashok Kumar*

**Supplementary Method**

**Datasets analyzed for the survival outcome**

Following gene expression datasets from GEO, EGA and TCGA were included for the survival analysis. E-MTAB-365, GSE11121, GSE12093, GSE12276, GSE1456, GSE16391, GSE16446, GSE16716, GSE17705, GSE17907, GSE19615, GSE20271, GSE2034, GSE20685, GSE20711, GSE21653, GSE25066, GSE2603, GSE26971, GSE2990, GSE31519, GSE3494, GSE37946, GSE42568, GSE45255, GSE4611, GSE46184, GSE48390, GSE4922, GSE5327, GSE61304, GSE65194, GSE6532, GSE69031, GSE7390, and GSE9195

**Somatic Genomic Alteration Analysis**

cBio Cancer Genomics Portal (http://cbioportal.org), an open access database, allows interactive analysis of multidimensional cancer genomics data and provides access to studies of more than 5,000 tumor samples from 20 cancer types (Gao et al., 2013). It contains genomic sequencing datasets from **5697** BC patients from **18** published studies. Genomic alterations summary ‘OncoPrint’ was used to summarize the genomic alterations of S1P metabolizing enzymes including *SPHK1*, *SPHK2*, *SGPP1*, *SGPP2*, *PLPP1*, *PLPP2*, *PLPP3*, and *SGPL1;* and S1P receptors *S1PR1*, *S1PR2*, *S1PR3*, *S1PR4*, *S1PR5* and *ITGAX* in BC datasets.

**References:**

1. Gao J, Aksoy BA, Dogrusoz U, Dresdner G, Gross B, Sumer SO, et al. Integrative analysis of complex cancer genomics and clinical profiles using the cBioPortal. Sci Signal. 2013;6: pl1. doi:10.1126/scisignal.2004088

**Supplementary Table 1. List of genes analyzed in the study**

| **Gene symbol:** | **Also known as** | **Affymetrix ID** | **Protein Name** |
| --- | --- | --- | --- |
| *SPHK1* | SPHK | 219257_s_at | Sphingosine Kinase 1 (SphK1) |
| *SPHK2* | SK2, SK-2, SPK 2, SPK-2 | 40273_at | Sphingosine Kinase 2 (SphK2) |
| *SGPP1* | SPP-1, SPPase1 | 223391_at | Sphingosine-1-phosphate phosphatase 1 (SGPP1) |
| *SGPP2* | SPPase2 | 226560_at | Sphingosine-1-phosphate phosphatase 2 (SGPP2) |
| *PLPP1* | LLP1a, LPP1, PAP-2a, PAP2, PPAP2A | 210946_at | Lipid Phosphate Phosphatase 1 (LPP1) |
| *PLPP2* | LPP2, PAP-2c, PAP2-g, PPAP2C | 212230_at | Lipid Phosphate Phosphatase 2 (LPP2) |
| *PLPP3* | Dri42, LPP3, PAP2B, PPAP2B, VCIP | 209529_at | Lipid Phosphate Phosphatase 3 (LPP3) |
| *SGPL1* | NPHS14, S1PL, SPL | 212321_at | Sphingosine-1-phosphate lyase (S1P lyase) |
| *S1PR1* | CD363, CHEDG1, D1S3362, ECGF1, EDG-1, EDG1, S1P1 | 204642_at | Sphingosine-1-phosphate receptor 1 (S1PR1) |
| *S1PR2* | AGR16, DFNB68, EDG-5, EDG5, Gpcr13, H218, LPB2, S1P2 | 227684_at | Sphingosine-1-phosphate receptor 2 (S1PR2) |
| *S1PR3* | EDG-3, EDG3, LPB3, S1P3 | 228176_at | Sphingosine-1-phosphate receptor 3 (S1PR3) |
| *S1PR4* | EDG6, LPC1, S1P4, SLP4 | 206437_at | Sphingosine-1-phosphate receptor 4 (S1PR4) |
| *S1PR5* | EDG8, Edg-8, S1P5, SPPR-1, SPPR-2 | 230464_at | Sphingosine-1-phosphate receptor 5 (S1PR5) |
| *ITGAX* | CD11C; SLEB6 | 210184_at | Integrin subunit alpha X |

**Supplementary Table 2^*^.** Correlation between mRNA expression of genes coding for S1P metabolic enzymes and survival outcome in breast cancer patients

| Gene | Survival | Patients | Hazard ratio | | | 95% CI | P value | |
| --- | --- | --- | --- | --- | --- | --- | --- | --- |
| *SPHK1* | RFS | 3951 | 0.9 | | | 0.81-1.01 | 0.079 | |
|  | OS | 1402 | 1.18 | | | 0.93-1.5 | 0.18 | |
|  | DMFS | 1746 | 1.31 | | | 1.04-1.66 | 0.023 | |
| *SPHK2* | RFS | 3951 | 0.53 | | | 0.48-0.6 | <1E-16 | |
|  | OS | 1402 | 0.76 | | | 0.6-0.96 | 0.019 | |
|  | DMFS | 1746 | 0.71 | | | 0.59-0.87 | 0.00087 | |
| *SGPP1* | RFS | 3951 | 0.66 | | | 0.57-0.77 | 1.6E-07 | |
|  | OS | 1402 | 0.59 | | | 0.43-0.81 | 0.00096 | |
|  | DMFS | 1746 | 0.79 | | | 0.57-1.1 | 0.17 | |
| *SGPP2* | RFS | 3951 | 0.94 | | | 0.8-1.01 | 0.43 | |
|  | OS | 1402 | 1.35 | | | 0.95-1.93 | 0.097 | |
|  | DMFS | 1746 | 1.66 | | | 1.09-2.52 | 0.017 | |
| *PLPP1* | RFS | 3951 | 0.81 | | | 0.73-0.91 | 0.00025 | |
|  | OS | 1402 | 0.76 | | | 0.61-0.96 | 0.018 | |
|  | DMFS | 1746 | 0.85 | | | 0.69-1.05 | 0.14 | |
| *PLPP3* | RFS | 3951 | 0.7 | | | 0.63-0.78 | 1.8e-10 | |
|  | OS | 1402 | 0.75 | | | 0.6-0.93 | 0.0086 | |
|  | DMFS | 1746 | |  | 0.87 | 0.7-1.08 | 0.21 |  |
| *PLPP2* | RFS | 3951 | |  | 0.92 | 0.82-1.03 | 0.16 |  |
|  | OS | 1402 | |  | 1.33 | 1.05-1.68 | 0.016 |  |
|  | DMFS | 1746 | |  | 1.3 | 1.06-1.6 | 0.011 |  |
| *SGPL1* | RFS | 3951 | |  | 0.92 | 0.81-1.04 | 0.2 |  |
|  | OS | 1402 | |  | 1.16 | 0.93-1.43 | 0.19 |  |
|  | DMFS | 1746 | |  | 0.77 | 0.61-0.97 | 0.031 |  |

**^*^**RFS = relapse-free survival; OS= Overall survival; DMFS = Distant metastasis-free survival

**Supplementary Table 3.** Correlation between mRNA expression of genes coding for S1P receptors and survival outcome in breast cancer patients

| Gene | Survival | Patients | Hazard ratio | 95% CI | P value |
| --- | --- | --- | --- | --- | --- |
| *S1PR1* | RFS | 3951 | 0.64 | 0.58-0.72 | 7.2e-16 |
|  | OS | 1402 | 0.83 | 0.67-1.03 | 0.094 |
|  | DMFS | 1746 | 0.87 | 0.71-1.07 | 0.19 |
| *S1PR2* | RFS | 3951 | 0.92 | 0.78-1.07 | 0.28 |
|  | OS | 1402 | 1.22 | 0.86-1.73 | 0.26 |
|  | DMFS | 1746 | 0.64 | 0.46-0.89 | 0.0077 |
| *S1PR3* | RFS | 3951 | 1.18 | 1.01-1.38 | 0.038 |
|  | OS | 1402 | 0.79 | 0.57-1.1 | 0.16 |
|  | DMFS | 1746 | 1.19 | 0.85-1.67 | 0.31 |
| *S1PR4* | RFS | 3951 | 0.81 | 0.73-0.91 | 0.00023 |
|  | OS | 1402 | 0.68 | 0.55-0.84 | 0.00042 |
|  | DMFS | 1746 | 0.91 | 0.75-1.11 | 0.37 |
| *S1PR5* | RFS | 3951 | 1.18 | 1.01-1.38 | 0.043 |
|  | OS | 1402 | 1.24 | 0.87-1.75 | 0.23 |
|  | DMFS | 1746 | 1.31 | 0.92-1.87 | 0.14 |

**^*^**RFS = relapse-free survival; OS= Overall survival; DMFS = Distant metastasis free survival

**Supplementary Table 4:** Correlation of *SGPP1* expression with *ITGAX* with in different cancer types

| **Cancer Type** | **Patient Nos.** | **Rho** | **P value** | **Adjusted p value** |
| --- | --- | --- | --- | --- |
| Breast invasive carcinoma -Basal | 191 | 0.53359741 | 1.90E-15 | 1.52E-14 |
| Uveal Melanoma | 80 | 0.49756212 | 2.66E-06 | 9.67E-06 |
| Ovarian serous cystadenocarcinoma | 303 | 0.49173744 | 7.47E-20 | 1.49E-18 |
| Thymoma | 120 | 0.46370581 | 9.62E-08 | 4.27E-07 |
| Stomach adenocarcinoma | 415 | 0.45401522 | 1.70E-22 | 6.81E-21 |
| Bladder Urothelial Carcinoma | 408 | 0.38168685 | 1.34E-15 | 1.34E-14 |
| Glioblastoma multiforme | 153 | 0.3670607 | 3.06E-06 | 1.02E-05 |
| Pancreatic adenocarcinoma | 179 | 0.35499778 | 1.08E-06 | 4.33E-06 |
| Liver hepatocellular carcinoma | 371 | 0.33516568 | 3.44E-11 | 2.29E-10 |
| Cholangiocarcinoma | 36 | 0.33178893 | 0.04805555 | 0.066283517 |
| Mesothelioma | 87 | 0.31676022 | 0.00279721 | 0.004475541 |
| Head and Neck squamous cell carcinoma -HPV+ | 98 | 0.30436279 | 0.00231126 | 0.003852099 |
| Colon adenocarcinoma | 458 | 0.27841371 | 1.34E-09 | 6.71E-09 |
| Lung squamous cell carcinoma | 501 | 0.27450457 | 4.12E-10 | 2.36E-09 |
| Rectum adenocarcinoma | 166 | 0.27372681 | 0.00035907 | 0.000797924 |
| Breast invasive carcinoma | 1100 | 0.26667521 | 2.30E-19 | 3.06E-18 |
| Cervical squamous cell carcinoma and endocervical adenocarcinoma | 306 | 0.25893808 | 4.44E-06 | 1.37E-05 |
| Breast invasive carcinoma -Her2 | 82 | 0.2418672 | 0.02858294 | 0.043676208 |
| Esophageal carcinoma | 185 | 0.23291573 | 0.00142017 | 0.002469854 |
| Breast invasive carcinoma -Lum B | 219 | 0.21822638 | 0.00115309 | 0.00219636 |
| Uterine Carcinosarcoma | 57 | 0.19490537 | 0.14625783 | 0.182822288 |
| Breast invasive carcinoma -Lum A | 568 | 0.18926556 | 5.58E-06 | 1.59E-05 |

**Supplementary Table 5: The Human Protein Atlas Data Analysis for *SGPP1* and *PLPP3***

| **Patient ID** | **Staining** | **Intensity** | **Quantification** | **IRS Score** |
| --- | --- | --- | --- | --- |
| ***SGPP1*** | | | | |
| 1910 | Medium | Moderate | >75% | **6** |
| 1688 | Medium | Moderate | >75% | **6** |
| 1874 | Medium | Moderate | 75%-25% | **4** |
| 2805 | Medium | Moderate | >75% | **6** |
| 2565 | Medium | Moderate | >75% | **6** |
| 2115 | Medium | Moderate | >75% | **6** |
| 4193 | Medium | Moderate | 75%-25% | **4** |
| 955 | Medium | Moderate | >75% | **6** |
| 145 | High | Strong | >75% | **9** |
| 2160 | ND | Negative | Non | **0** |
| 915 | ND | Negative | Non | **0** |
| 2428 | ND | Negative | Non | **0** |
| ***PLPP3*** | | | | |
| 1785 | Medium | Moderate | 75%-25% | **4** |
| 2091 | Medium | Moderate | >75% | **6** |
| 3257 | Medium | Moderate | 75% | **4** |
| 1775 | Medium | Moderate | 75%-25% | **4** |
| 2565 | LOW | Weak | 75%-25% | **4** |
| 2252 | LOW | Weak | 75%-25% | **4** |
| 1916 | LOW | Moderate | <25% | **2** |
| 3535 | LOW | Moderate | <25% | **2** |
| 1874 | ND | Negative | Non | **0** |
| 1939 | ND | Negative | Non | **0** |
| 1910 | ND | Negative | Non | **0** |
| 2428 | ND | Weak | <25% | **1** |

*ND= Not Detected


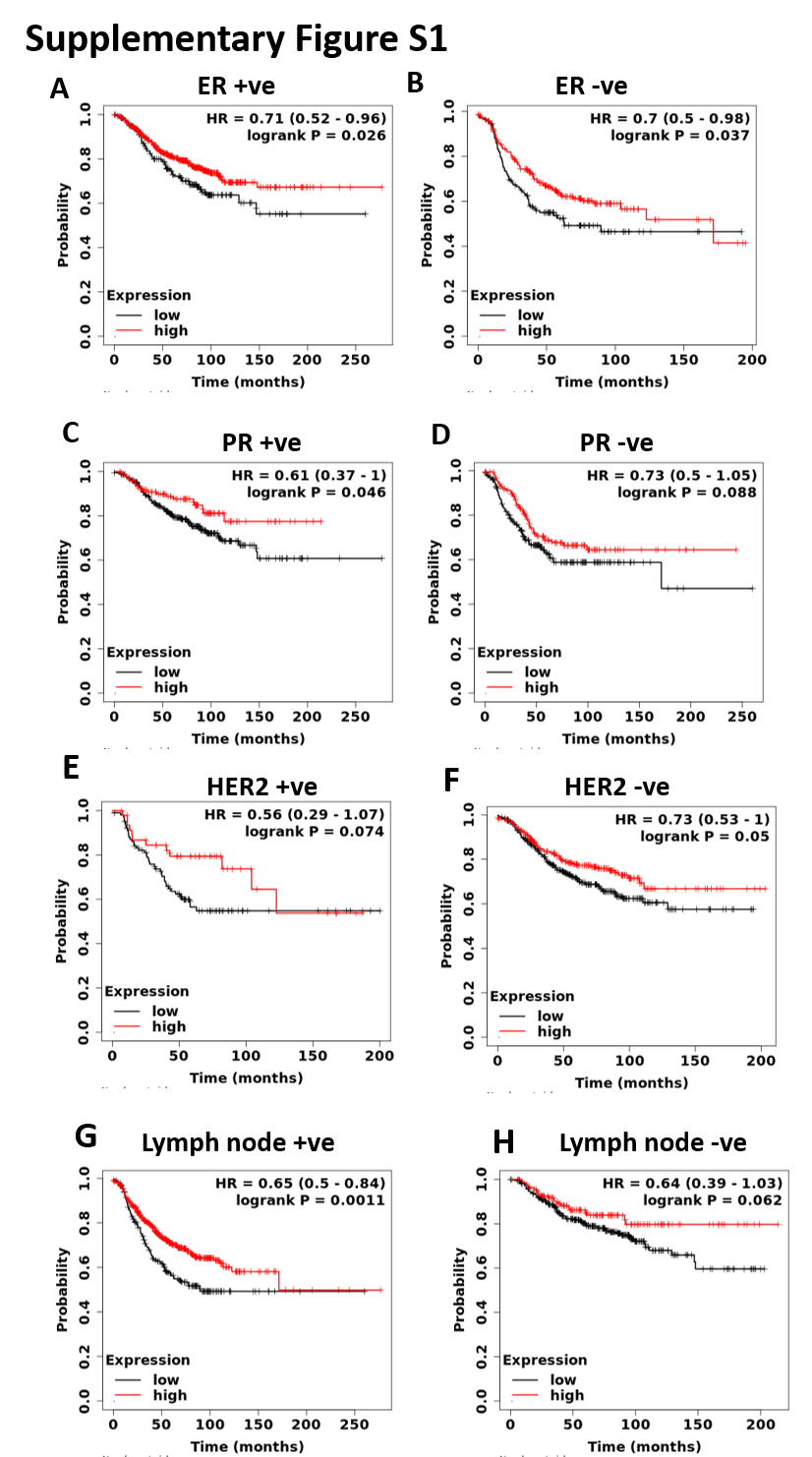


**Supplementary Figure S1A-H.** Expression of SGPP1 is significantly associated with RFS of ER+/ER-, PR+, HER2- and lymph node +ve status in BC patients. A-H, the Kaplan-Meier survival curves (RFS) were plotted for SGPP1 for hormone receptor (ER+/ER-, PR+/PR- and HER2+/HER2) and lymph node status of breast cancer patients.


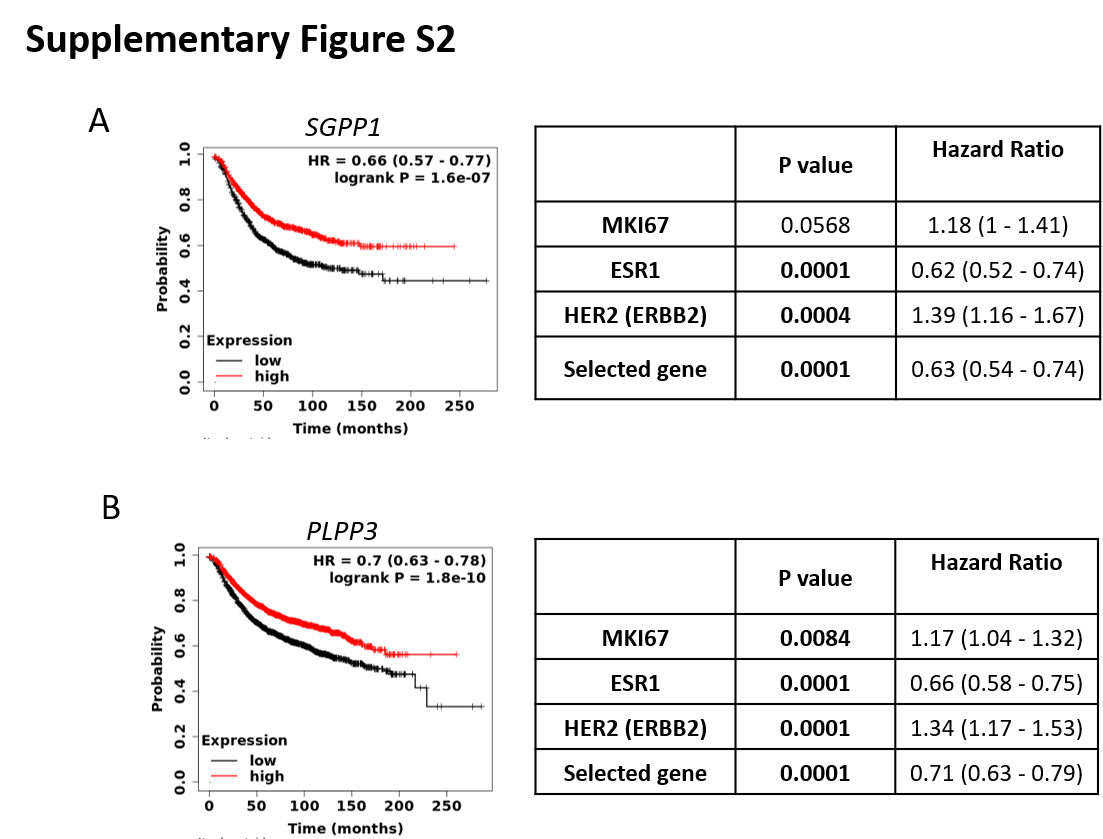


**Supplementary Figure S2:** The Kaplan-Meier survival curves were plotted for *SGPP1* (A) and *PLPP3* (B) for multivariate analysis with selected variables like MKI67, ESR1 and ERB2 and results showed highly association with RFS of BC patients.


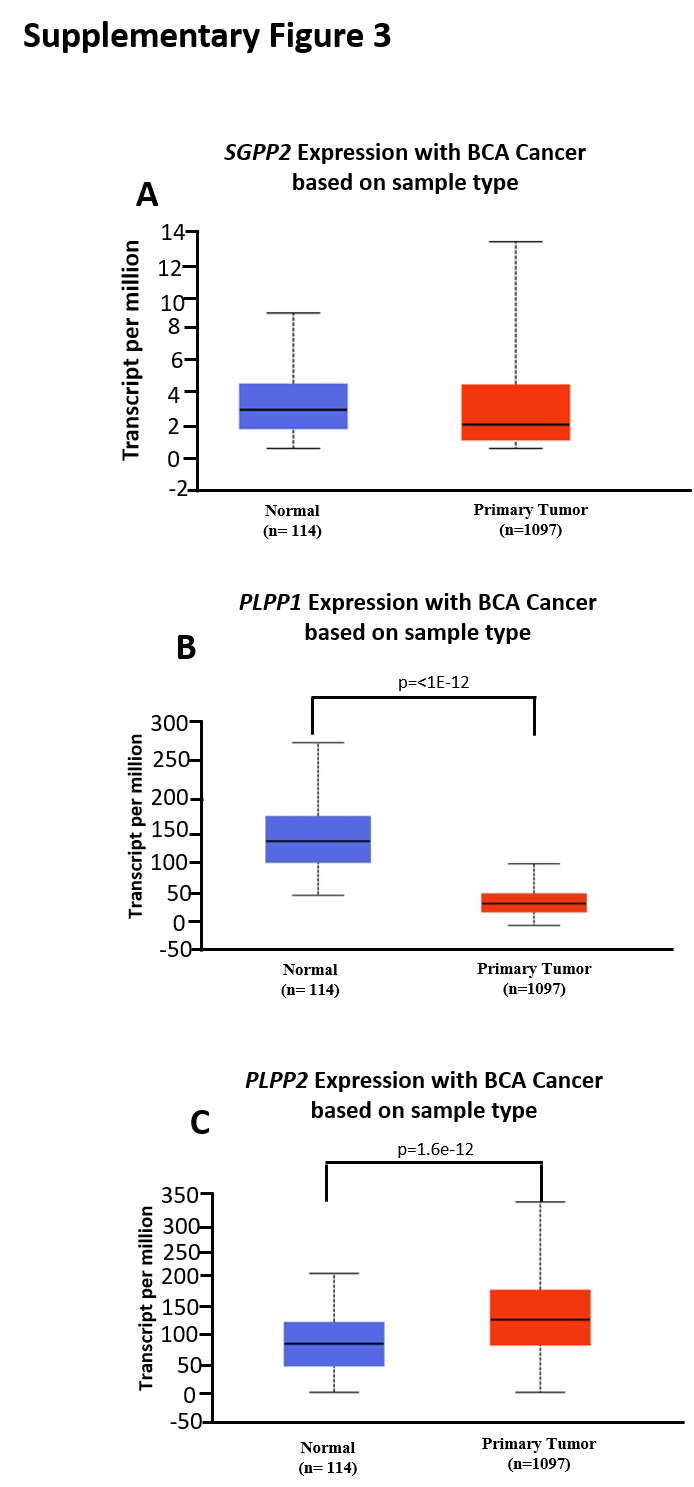


**Supplementary Figure S3.** mRNA expression of *SGPP2* **(A)**, *PLPP1* **(B)** and *PLPP2* **(C)** was analyzed in the normal breast tissue (N=114) and primary breast tumors(N=1097) from invasive breast carcinoma patients from the publicly available UALCAN database. Data is shown as average transcript per million; P value has been shown on the primary tumors vs normal tissues in the corresponding genes.


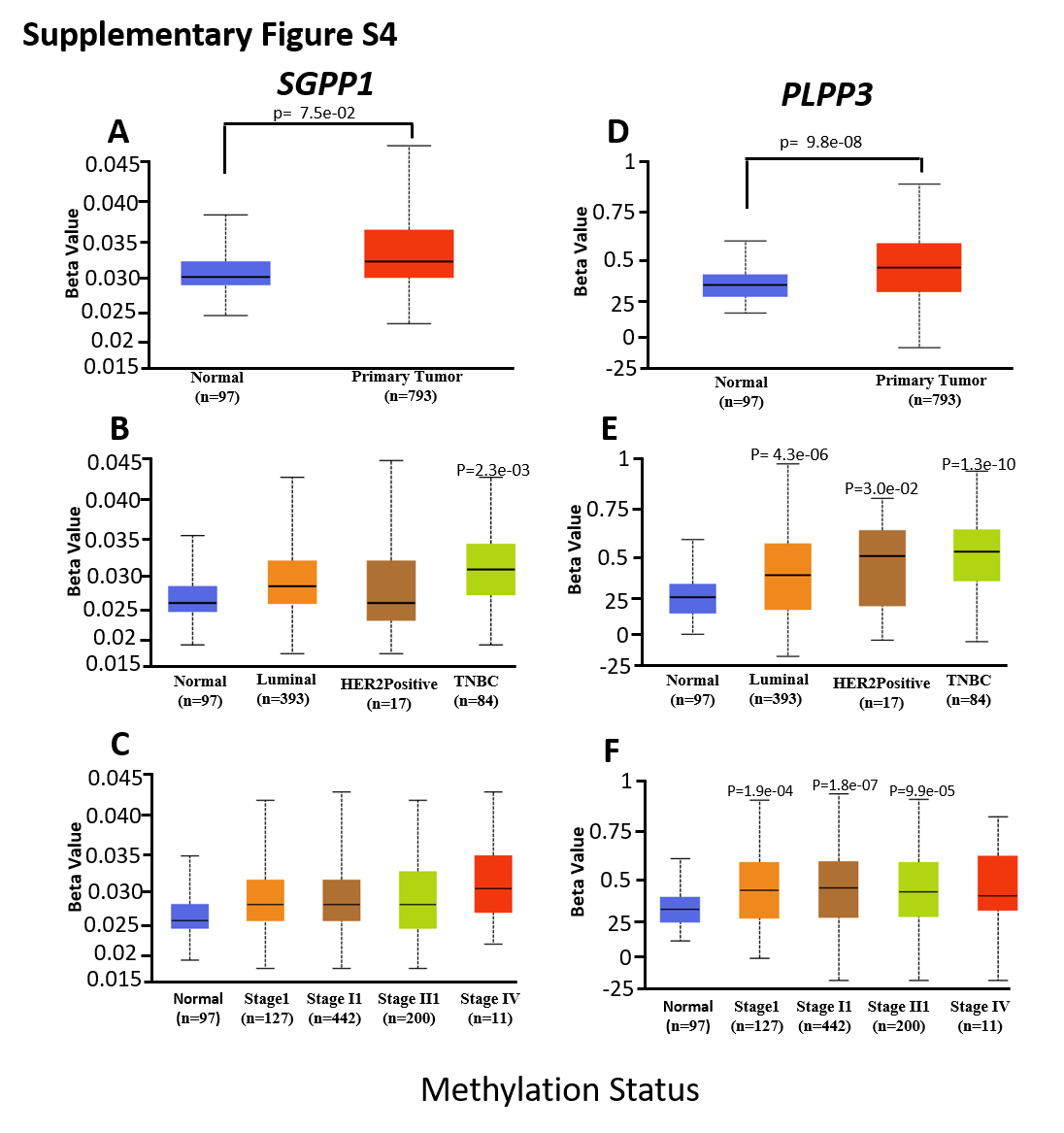


**Supplementary Figure S4.** Promoter methylation status was analyzed for *SGPP1* and *PLPP3* genes in the normal breast tissue (N=97) and BC tissue (N=793) patients from the publicly available UALCAN database. **A-C,** *SGPP1* and **D-F**, *PLPP3*; **A & D,** Normal breast tissue Vs Tumors from invasive BC patients; **B & E**, Normal tissue vs tumor tissue from different intrinsic subtypes; and **C & F,** Normal tissue vs tumor tissues from different stages of BC. Data is shown as average transcript per million; P value has been shown on the primary tumors vs normal tissues in the corresponding genes.

**
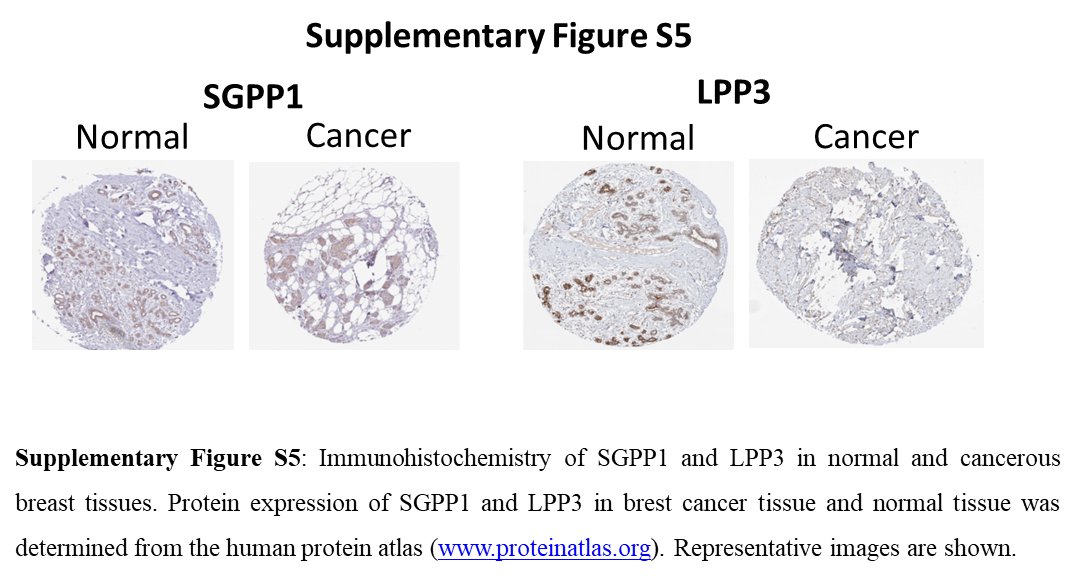
**

**
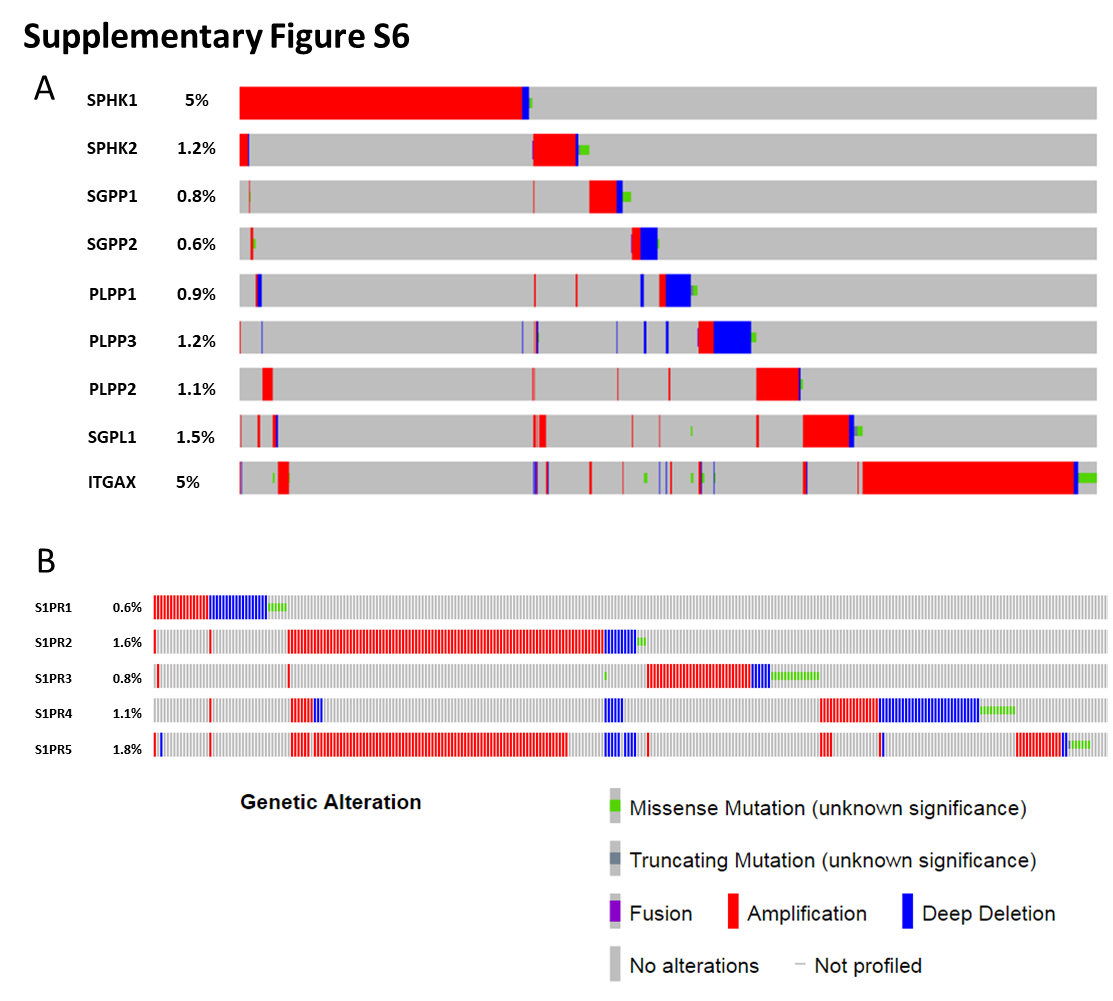
**

**Supplementary Figure S6.** Genetic alterations in genes coding S1P metabolizing enzymes **(A)** and S1P receptors **(B)** were analyzed in BC patients (N= 7450) by cBioportal database. Data is shown in the Oncoprint format.


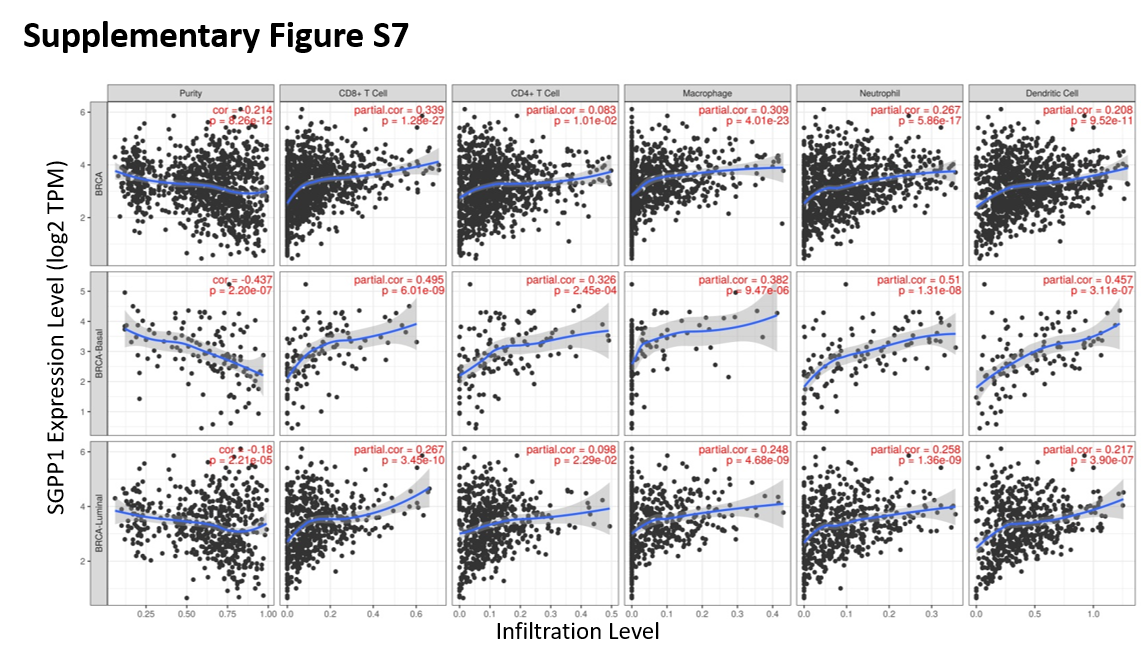


**Supplementary Figure S7.** Correlation of *SGPP1* expression with immune infiltration level with invasive breast carcinoma, basal and luminal subtypes, *SGPP1* expression was significant correlated with infiltrating levels of CD4+ and CD8+ T cells, macrophages, neutrophils and dendritic cells in TNBC (basal-like subtype).


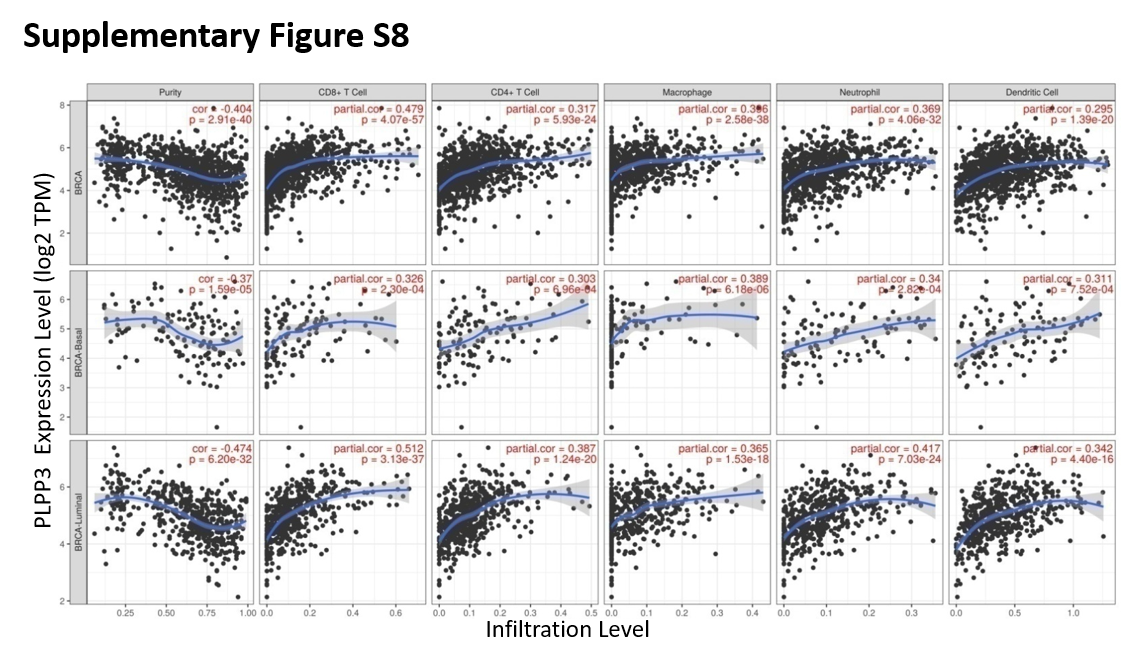


**Supplementary Figure S8.** Correlation of *PLPP3* expression with tumor-infiltrating immune cells in different intrinsic subtypes of BC patients was analyzed by *TIMER*. *PLPP3* expression is significant correlated with the numbers of tumor infiltrating CD4+ and CD8+ T cells, dendritic cells, neutrophils and macrophages in basal-like and luminal subtypes.


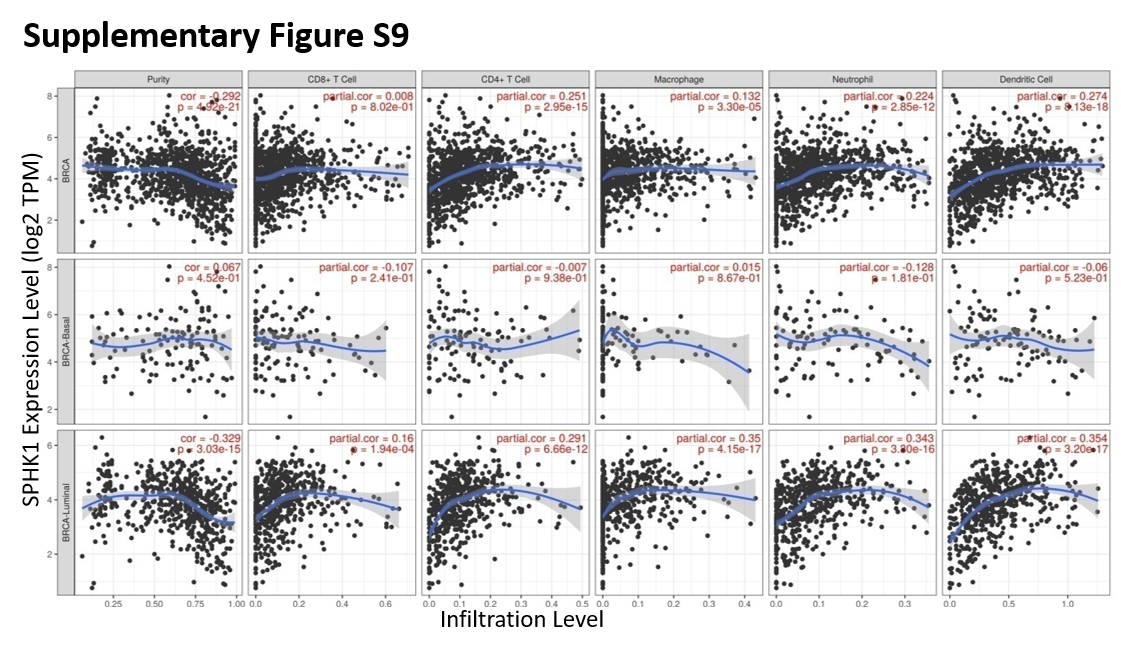


**Supplementary Figure S9.** Correlation of *SPHK1* expression with tumor-infiltrating immune cells in different intrinsic subtypes of BC patients was analyzed by *TIMER*. *SPHK1* expression is significant correlated with the numbers of tumor infiltrating CD4+ and CD8+ T cells, dendritic cells, neutrophils and macrophages in the luminal subtypes.


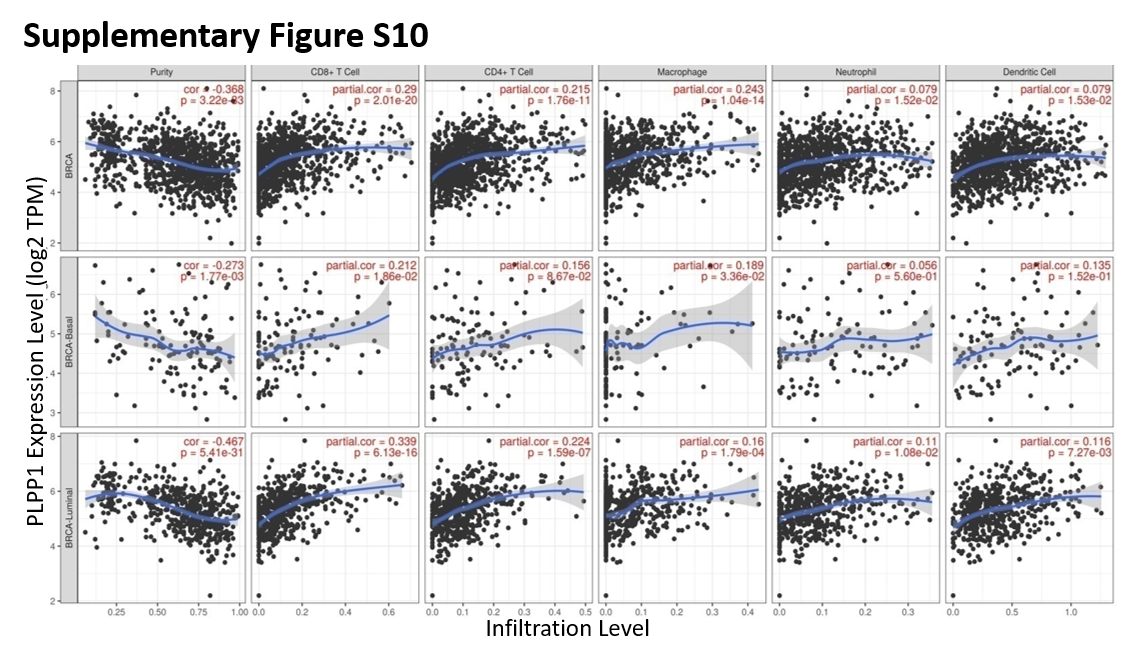


**Supplementary Figure S10.** Correlation of *PLPP1* expression with tumor-infiltrating immune cells in different intrinsic subtypes of BC patients was analyzed by *TIMER*. *PLPP1* expression is only significant correlated with the numbers of tumor infiltrating CD8+ T cells in the luminal subtypes.


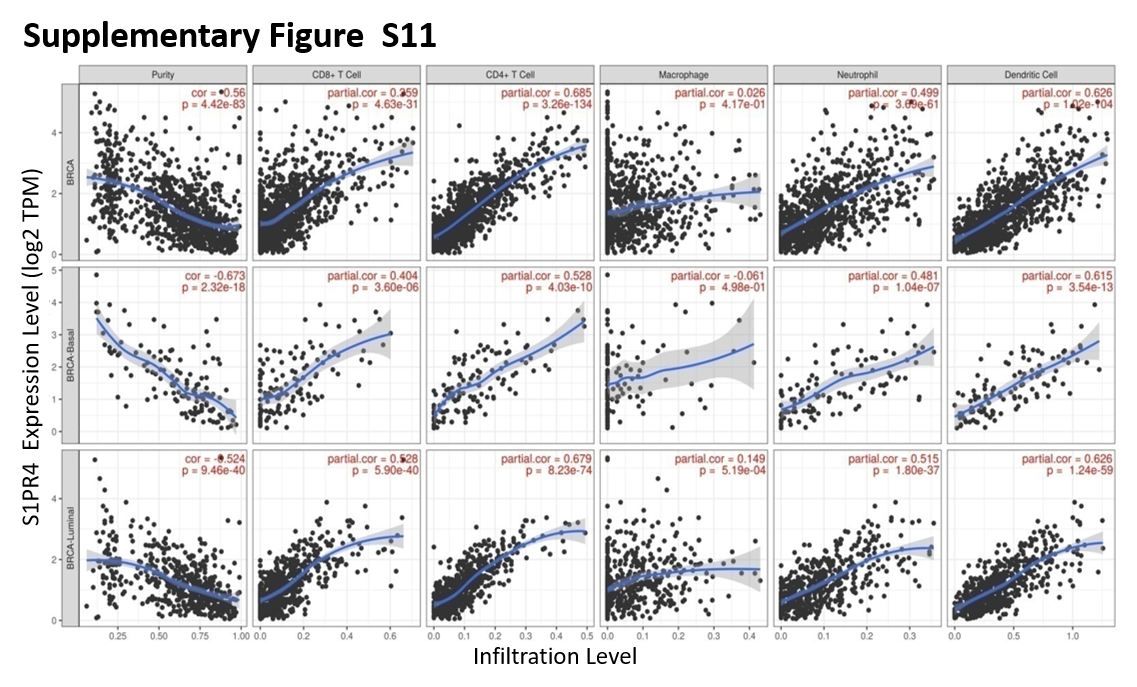


**Supplementary Figure S11.** Correlation of *S1PR4* expression with immune infiltration level with invasive breast carcinoma, basal and Luminal, where S1PR4 showed the strongest correlation between expression of *S1PR4* and tumor infiltrating immune CD4+ T cells, CD8+ T cells, neutrophils and dendritic cells, except macrophages in all the subtypes of BC.


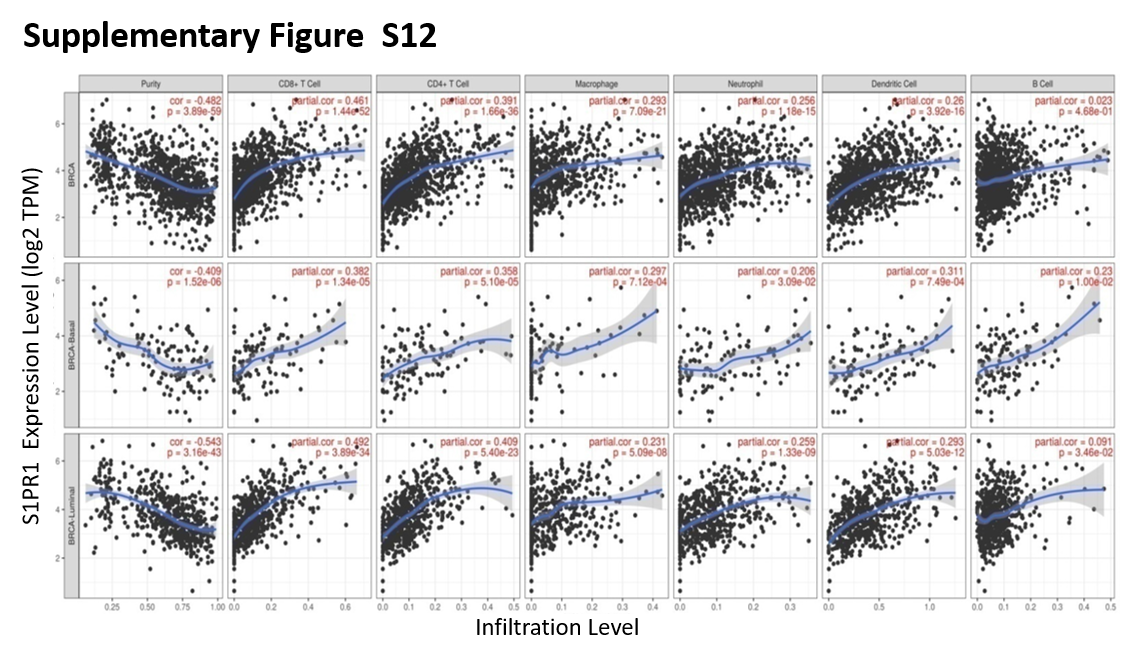


**Supplementary Figure S12.** Correlation of *S1PR1* expression with tumor-infiltrating immune cells in different intrinsic subtypes of BC patients was analyzed by *TIMER*. *S1PR1* expression is significant correlated with the numbers of tumor infiltrating B cells, CD4+ and CD8+ T cells, dendritic cells, neutrophils, macrophages in the basal and luminal subtypes.


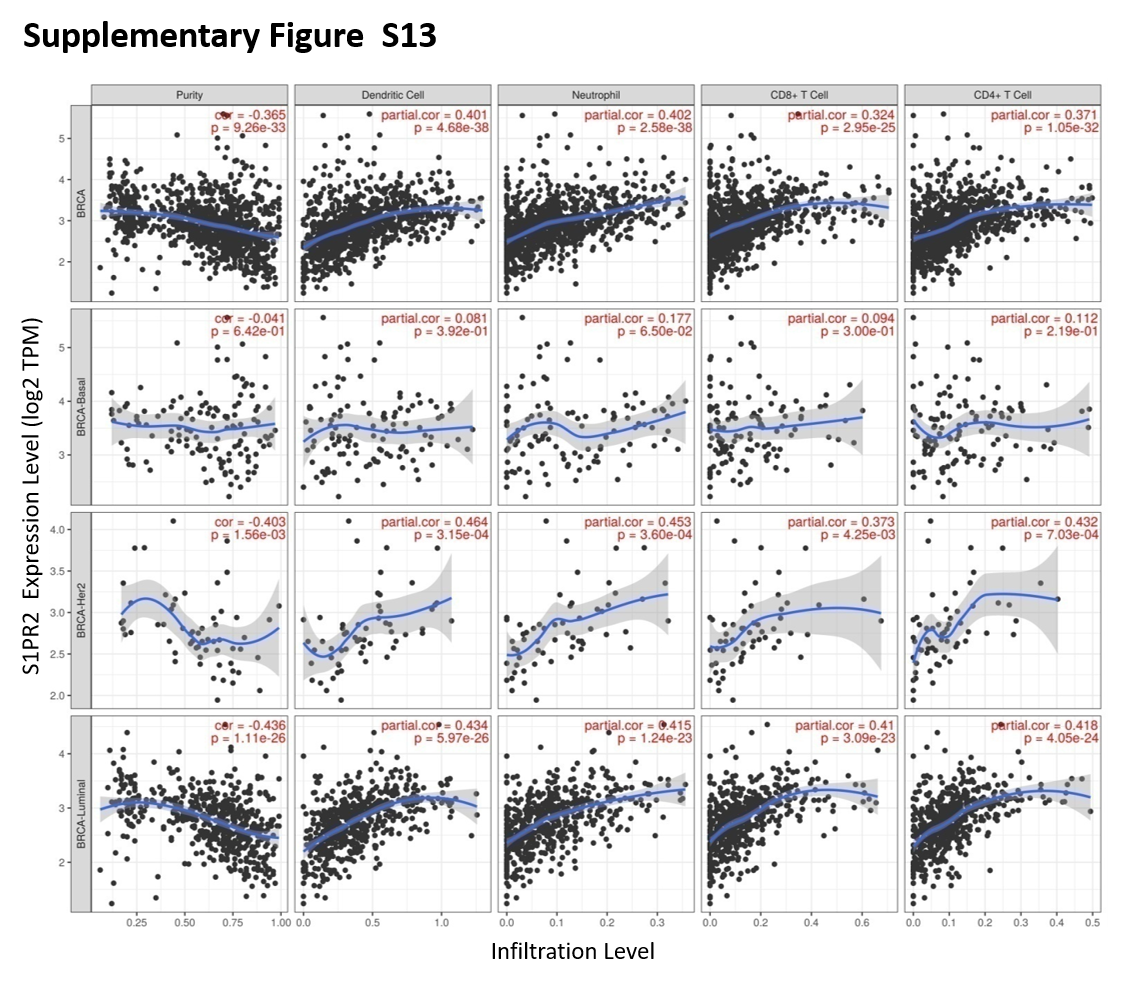


**Supplementary Figure S13.** Correlation of *S1PR2* expression with tumor-infiltrating immune cells in different intrinsic subtypes of BC patients was analyzed by *TIMER*. *S1PR2* expression is significantly correlated with the numbers of tumor infiltrating CD4+ and CD8+ T cells, dendritic cells, neutrophils, in the luminal and HER2 +ve subtypes.


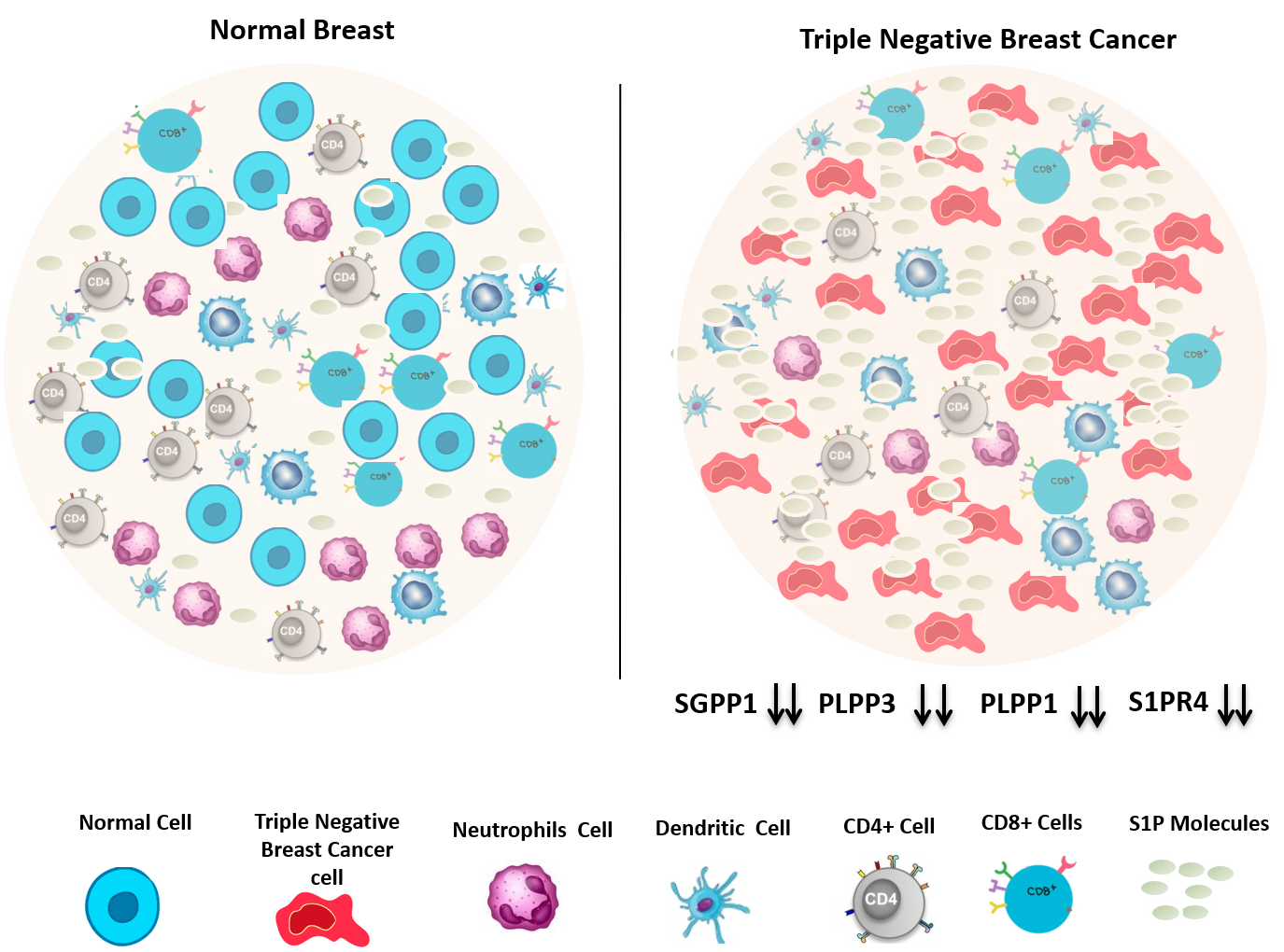


**Supplementary Figure S14.** Expression of genes coding for S1P catabolizing enzymes namely *SGPP1*, *PLPP1* and *PLPP3* and *S1PR4* is downregulated in tumor tissue from triple-negative breast cancer (BC) patients, possibly resulting into accumulation of sphingosine-1-phosphate (S1P). BC patients with low expression of *SGPP1*, *PLPP1* and *PLPP3* exhibit poor survival outcome. Expression of *SGPP1*, *PLPP1* and *PLPP3* positively correlate with number of infiltrating immune cells, including dendritic cells, CD4+ T cells, CD8+ T cells, macrophage and neutrophils.
